# A Single-cell and Spatially Resolved Cell Atlas of Human Esophageal Squamous Cell Carcinoma

**DOI:** 10.1093/gpbjnl/qzaf095

**Published:** 2025-11-04

**Authors:** Yong Shi (石勇), Ke An (安珂), Yu Qi (齐宇), Xinhan Zhang (张鑫涵), Yueqin Wang (王月琴), Xuran Zhang (张旭冉), Shaoxuan Zhou (周韶轩), Ouwen Li (李欧文), Yanan Song (宋亚楠), Jiayi Zhou (周佳熠), Yue Du (杜玥), Mingyang Hou (候明阳), Yun-Gui Yang (杨运桂), Xin Tian (田鑫)

**Affiliations:** Department of Pharmacy, the First Affiliated Hospital of Zhengzhou University, Zhengzhou 450052, China; Department of Pharmacy, the First Affiliated Hospital of Zhengzhou University, Zhengzhou 450052, China; Henan Key Laboratory of Precision Clinical Pharmacy, Zhengzhou University, Zhengzhou 450052, China; Department of Thoracic Surgery, the First Affiliated Hospital of Zhengzhou University, Zhengzhou 450052, China; Department of Pharmacy, the First Affiliated Hospital of Zhengzhou University, Zhengzhou 450052, China; Department of Pharmacy, the First Affiliated Hospital of Zhengzhou University, Zhengzhou 450052, China; Henan Key Laboratory of Precision Clinical Pharmacy, Zhengzhou University, Zhengzhou 450052, China; Department of Pharmacy, the First Affiliated Hospital of Zhengzhou University, Zhengzhou 450052, China; Department of Pharmacy, the First Affiliated Hospital of Zhengzhou University, Zhengzhou 450052, China; Department of Pharmacy, the First Affiliated Hospital of Zhengzhou University, Zhengzhou 450052, China; Department of Thoracic Surgery, the First Affiliated Hospital of Zhengzhou University, Zhengzhou 450052, China; China National Center for Bioinformation, Beijing 100101, China; Beijing Institute of Genomics, Chinese Academy of Sciences, Beijing 100101, China; University of Chinese Academy of Sciences, Beijing 100101, China; Department of Pharmacy, the First Affiliated Hospital of Zhengzhou University, Zhengzhou 450052, China; Henan Key Laboratory of Precision Clinical Pharmacy, Zhengzhou University, Zhengzhou 450052, China; Department of Pharmacy, the First Affiliated Hospital of Zhengzhou University, Zhengzhou 450052, China; China National Center for Bioinformation, Beijing 100101, China; Beijing Institute of Genomics, Chinese Academy of Sciences, Beijing 100101, China; University of Chinese Academy of Sciences, Beijing 100101, China; Department of Pharmacy, the First Affiliated Hospital of Zhengzhou University, Zhengzhou 450052, China; Henan Key Laboratory of Precision Clinical Pharmacy, Zhengzhou University, Zhengzhou 450052, China

**Keywords:** Esophageal squamous cell cancer, Tumor microenvironment, Spatial transcriptomics, Single-cell sequencing, Tumor heterogeneity

## Abstract

Tumor heterogeneity and the suppressive microenvironment are key challenges that limit the effectiveness of cancer treatment. In this study, we systematically elucidated the molecular characteristics and mechanisms underlying the suppressive immune microenvironment using a combination of single-cell RNA sequencing, spatial transcriptomics, and metabolomics for a series of human esophageal squamous cell carcinoma and matched non-tumor tissues. We found that COL17A1^+^ epithelial cells exhibited greater malignancy, characterized by the accumulation of triglycerides and phosphocholine. We also identified a tumor-specific POSTN^+^ fibroblast subgroup. We identified a unique epithelial-fibroblast niche with low infiltration of effector immune cells and substantial lipid enrichment, composed of POSTN^+^ fibroblasts and COL17A1^+^ epithelial cells, in which their crosstalk contributed to tumor progression. We confirmed that the *INHBA*/*TP63* axis played a key role in mediating the regulation of COL17A1^+^ tumor cells by POSTN^+^ fibroblasts. Our findings provide new insights into the characteristics of the tumor microenvironment and the crosstalk between tumor cells and fibroblasts, offering valuable multi-omics data for elucidating tumor progression mechanisms.

## Graphical abstract

**Figure:**
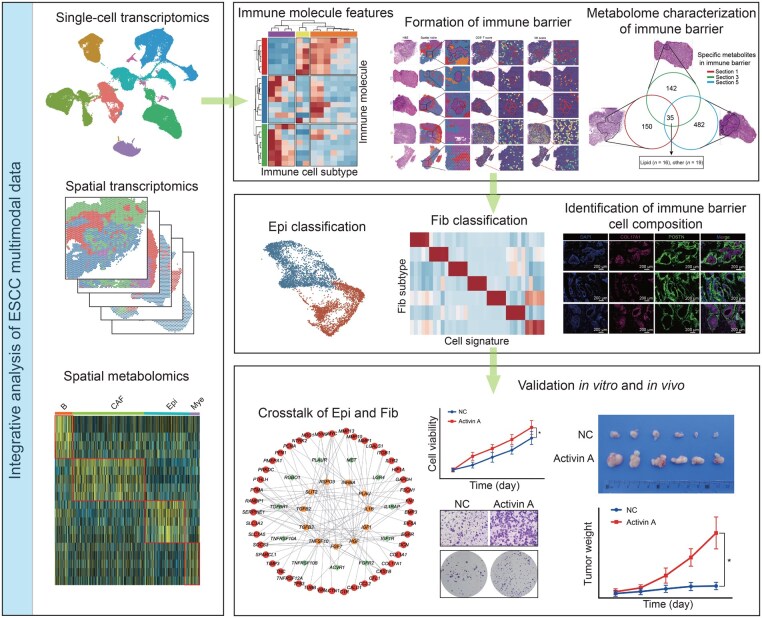

## Introduction

Esophageal cancer is the seventh most prevalent malignancy and the sixth leading cause of tumor-associated death globally [1]. Esophageal squamous cell cancer (ESCC) accounts for ∼ 90% of these cases [2]. Patients with early-stage ESCC generally receive effective treatment after excision. However, after ESCC reaches an advanced stage or metastasizes, most patients succumb to the disease. Immune checkpoint blockade (ICB) strategies are used for treating advanced and metastatic diseases, including ESCC [3,4]. Although ICB strategies have several benefits, their effectiveness is often limited by tumor microenvironment (TME) heterogeneity [5]. Therefore, the molecular characteristics of the TME need to be elucidated.

The combined analysis of single-cell data and spatial data not only provides transcriptional profiles of individual cells but also preserves their spatial information, offering significant advantages in investigating the heterogeneity of solid tumors and the features of the TME [6,7]. Integrative analysis strategies have been widely used in various types of cancer, resolving the interactions of key cellular subpopulations associated with tumor progression, metastasis, and resistance to treatment [8–10]. Several studies on ESCC have focused on identifying critical protumor molecular events [11–13] and characterizing the distribution of immune and stromal cells in metastatic and nonmetastatic samples [14]. Therefore, further assessment of the cell interactions in the TME and how these interactions affect the spatial architecture of the microenvironment is essential.

Besides single-cell and spatial data, additional omics information is necessary to gain comprehensive insights into the TME. Disorders of cellular interaction and metabolism are two key determinants that significantly affect tumor immune evasion, invasion, metastasis, and resistance to therapy [15,16]. Variations in metabolism are closely related to spatial heterogeneity [17]. The development of matrix-assisted laser desorption/ionization mass spectrometry imaging (MALDI-MSI) has provided a key method for examining the spatial features of metabolites in heterogeneous diseased tissues [18], thus enabling the delineation of the metabolic profile in the TME. Therefore, the integration of multi-omics data may provide a comprehensive understanding of the mechanisms underlying tumor progression and the reprogramming of the TME.

In this study, by integrating single-cell transcriptomics, spatial transcriptomics (ST), and spatial metabolomics (SM), we found important molecular events in the suppressive immune microenvironment. We also showed the heterogeneity and molecular features of tumor cells and fibroblasts. We identified a spatial structure composed of specific tumor cells and fibroblasts in ESCC characterized by a decrease in immune cell infiltration, emphasizing its importance in tumor immune evasion. We also systematically deciphered the mutual regulatory network and functions of tumor cells and fibroblasts within this structure. Overall, the findings of this study improved our understanding of the complexity of the TME in ESCC.

## Results

### Single-cell and spatial multi-omics profiling of ESCC

**Figure 1:**
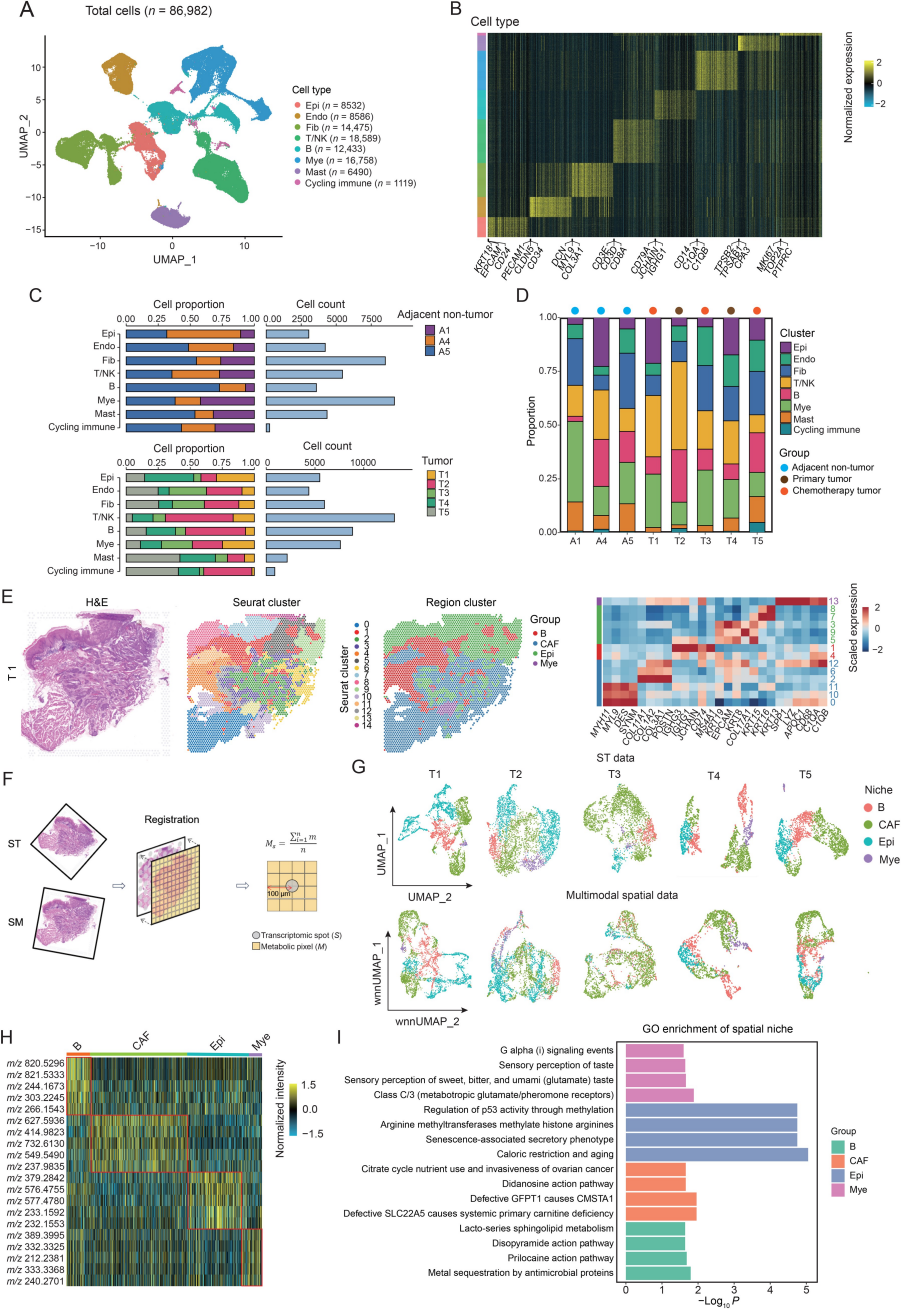
Single-cell and multi-omics cell atlas in ESCC **A**. UMAP plots of total cells labeled by main cell types from paired adjacent non-tumor and tumor tissues in ESCC. **B**. Heatmap of the top 50 cell-type-specific genes for each major cell type in ESCC. **C**. Bar plots showing the proportion of eight cell types stratified by tumor sample, tissue type, and total cell count. **D**. Bar plots showing the cell proportion of major cell types across eight samples. **E**. Left: H&E staining of tissue sections; unbiased clustering of ST spots and four spatial niche clusters. Right: heatmap showing the expression of marker genes for four spatial niches. **F**. Integrated analysis workflow of ST and MALDI-MSI data. **G**. UMAP plots of ST data and WNN-UMAP plots of multimodal data, both labeled by four spatial niches. **H**. Heatmap displaying the top 10 differentially abundant metabolites for each spatial niche. **I**. Bar plots showing the top 4 GO terms enriched for niche-specific metabolites. UMAP, Uniform Manifold Approximation and Projection; NK, natural killer; H&E, hematoxylin and eosin; CAF, cancer-associated fibroblast; Mye, myeloid cell; Epi, epithelial cell; Endo, endothelial cell; Cycling immune, cycling immune cell; B, B cell; T/NK, T/NK cell; Fib, fibroblast; ST, spatial transcriptomics; MALDI-MSI, matrix-assisted laser desorption/ionization mass spectrometry imaging; WNN, weighted nearest neighbor; GO, Gene Ontology.

To establish a single-cell and spatial multi-omics atlas of ESCC, we recruited five ESCC patients (Table S1). The integrated analysis workflow is shown in Figure S1A. Single-cell RNA sequencing (scRNA-seq) was performed on five tumor tissues and three paired adjacent non-tumor tissues. ST [19] and MALDI-MSI analyses [20] were conducted on consecutive sections from the five tumor tissue samples. A total of 92,526 cells were obtained from scRNA-seq. After quality control, 86,982 high-quality cells were retained, with an average sequencing depth of 1243 genes per cell (Figure S1B and C). The data were integrated using the robust principal component analysis (RPCA) method, which effectively minimized batch effects (Figure S1D and E). Twenty cell clusters were identified via unsupervised clustering (Figure S1F). Based on the mRNA expression of the known cell-specific signature genes, eight major cell clusters were annotated across tumor and adjacent non-tumor tissues, including epithelial cells (*KRT18*, *EPCAM*, and *CD24*), endothelial cells (*PECAM1*, *CLDN5*, and *CD34*), fibroblasts (*DCN*, *MYL9*, and *COL3A1*), T/natural killer (NK) cells (*CD3E*, *CD3D*, and *CD8A*), B cells (*CD79A*, *JCHAIN*, and *IGHG1*), myeloid cells (*CD14*, *C1Q1*, and *C1QB*), mast cells (*TPSB2*, *TPSAB1*, and *CPA3*), and cycling immune cells (*MKI67*, *TOP2A*, and *PTPRC*) (**Figure 1**A and B, Figure S1G). The results of the cell type proportion analysis reflected the degree of variation in cell abundance among the ESCC samples, highlighting the heterogeneity and complexity of interpatient tumor tissues (Figure 1C and D).

For ST data, we obtained valid transcriptomic data from 3653, 3790, 2856, 1710, and 2169 spots in five samples, with median depths of 8817, 8024, 1968, 9414, and 3219 unique molecular identifier (UMI) counts per spot and 3130, 2877, 1071, 3320, and 1473 genes per spot, respectively (Figure S2A and B). Integrated analysis of ST and scRNA-seq revealed spatially restricted distribution patterns for distinct cell types (Figure S2C). By conducting unbiased clustering via Seurat, the spots were categorized into 15, 9, 9, 7, and 9 clusters for the five sections, respectively (Figure 1E, Figure S2D). Based on marker gene expression from these spatial clusters, we annotated all sections into four regions: epithelial cells, B cells, cancer-associated fibroblasts (CAFs), and myeloid niches (Figure 1E, Figure S2D).

For MALDI-MSI analyses, 1474 metabolites were detected across five tumor samples. To further integrate ST with SM data, we adopted a novel workflow involving two major steps: (1) alignment of ST and SM images and (2) calculation of the metabolic intensity for each spatial spot (Figure 1F). We combined ST and SM data to create multimodal data and mapped it to the weighted nearest-neighbor-Uniform Manifold Approximation and Projection (WNN-UMAP) space (Figure 1G). The four spatial niches also generated separate clusters in the WNN-UMAP space, confirming the effectiveness and robustness of our integration methods (Figure 1G). By conducting a differential abundant metabolite analysis, we identified spatial niche-specific metabolites (Figure 1H). Further functional enrichment analysis revealed that metabolomics varied across different spatial niches, such as significant enrichment of sphingolipid metabolism in the B cell niche, arginine methyltransferases (RMTs) that methylate histone arginine in the epithelial cell niche, the citrate cycle in the fibroblast niche, and metabotropic glutamate in the myeloid niche (Figure 1I). To summarize, we constructed a single-cell and spatial multi-omics cell atlas of ESCC.

### The immune landscape of ESCC

**Figure 2:**
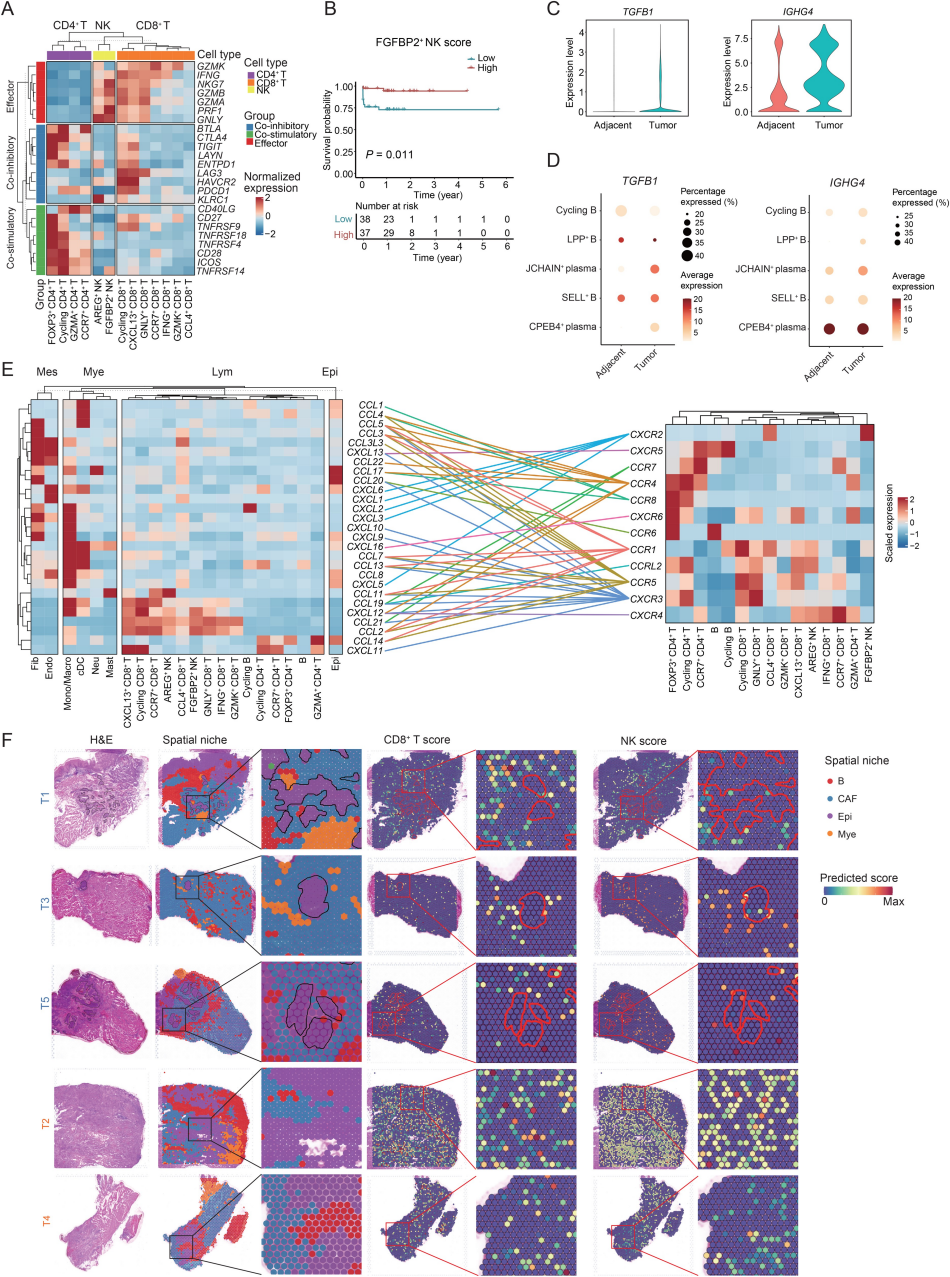
The immune landscape of ESCC **A**. Scaled mean normalized mRNA expression heatmap of genes related to cytotoxicity, co-inhibitory, and co-stimulatory molecules across T cells and NK cells subsets in scRNA-seq data. **B**. Kaplan–Meier survival curve for FGFBP2^+^ NK cells from a TCGA cohort of 75 ESCC patients. **C**. Violin plots of *TGFB1* and *IGHG4* comparing B cells in adjacent *versus* tumor tissues based on scRNA-seq data. **D**. Dot plots showing the mRNA expression of *TGFB1* and *IGHG4* between non-tumor and tumor tissues among B cell subpopulations. **E**. Left: scaled mean normalized mRNA expression heatmap of chemokine genes in distinct cell types from scRNA-seq data. Right: scaled mean normalized mRNA expression heatmap of chemokine receptor genes in distinct lymphocyte cell types from scRNA-seq data. Chemokines and matching receptors were linked by color lines. **F**. Left: H&E staining and spatial clusters with annotated tumor region (solid lines) across five tumor samples. Right: spatial feature plots showing the enrichment scores of CD8^+^ T and NK cells across five tumor samples. A *P* < 0.05 is considered statistically significant. Statistical significance was assessed using a two-sided log-rank test in (B). scRNA-seq, single-cell RNA sequencing; TCGA, The Cancer Genome Atlas; Lym, lymphoid cell; Mes, mesenchymal cell; Neu, neutrophil; Mono/Maro, monocyte/macrophage.

The occurrence and development of tumors are closely related to the TME [21]. To gain insights into the spatial organization of the immune microenvironment in ESCC, we first conducted subpopulation clustering of immune cells. After unbiased clustering, the total immune cells could be split into five major compartments and further annotated as 19 distinct subpopulations, each distinguished by specific marker genes (Figure S3A–C). The expression of known key immune genes was evaluated across immune cell subtypes (**Figure 2**A). Three CD8^+^ T cell subsets (CXCL13^+^ CD8^+^ T cells, GNLY^+^ CD8^+^ T cells, and cycling CD8^+^ T cells) expressed high levels of immune effector molecules and exhibited high expression of immune inhibitory molecules, reflecting immune exhaustion in the TME (Figure 2A). Both FGFBP2^+^ NK and AREG^+^ NK cells presented high expression of immune effector molecules. However, FGFBP2^+^ NK cells expressed inhibitory molecules at lower levels and had good prognostic value, suggesting that they may be in an immune-activated state (Figure 2A and B).

Our multi-omics data identified that B cells constitute a major component of the TME (Figures S2D and S3A), suggesting that B cells may play key roles in reshaping the TME. Another study reported that an important B cell subpopulation, known as regulatory B cells (Bregs), can weaken the immune response to tumors by secreting TGFB1 or IGHG4 [22,23]. Our data revealed a significant increase in the expression of *TGFB1* and *IGHG4* in B cells within tumor tissues (Figure 2C). Compared to that in adjacent non-tumor tissues, the expression of *TGFB1* was significantly greater in CPEB4^+^ plasma cells and JCHAIN^+^ plasma cells within tumor tissues, and *IGHG4* expression was markedly elevated in LPP^+^ B cells and JCHAIN^+^ plasma cells in tumor tissues (Figure 2D). Spatial analysis also revealed high mRNA expression of *IGHG4* and *TGFB1* in the B cell niche (Figure S3D). Additionally, several classic immunosuppressive molecules also occur in B cells (Figure S3E). Hence, our findings suggest that B cells are involved in the generation of an inhibitory microenvironment.

Potential mechanisms underlying the recruitment of tumor-infiltrating lymphocytes were subsequently assessed by analyzing chemokine and receptor expression in the scRNA-seq data (Figure 2E). Half of the detected chemokine receptors were expressed predominantly in regulatory T cells (Tregs) (Figure 2E). Most chemokine-recruiting Tregs were highly expressed in fibroblasts, endothelial cells, monocytes/macrophages, and classic dendritic cells (cDCs), suggesting that these cells are responsible for recruiting Tregs (Figure 2E). FGFBP2^+^ NK cells, which are important antitumor effector cells, expressed only one chemokine receptor from the chemokine pool we detected, implying insufficient recruitment of FGFBP2^+^ NK cells in the TME and limiting their antitumor effects (Figure 2E).

Major antitumor immune cell types, including CD8^+^ T cells and NK cells, were mapped to the ST data. The quantitative analysis revealed a decrease in the colocalization of tumor cells with CD8^+^ T or NK cells in tumor samples 1, 3, and 5 (Figure S3F and G). A unique spatial barrier was formed in tumor samples 1, 3, and 5, in which CAFs enveloped tumor cells (Figure 2F). Therefore, we speculated that the reduction in immune cell infiltration might be related to the formation of spatial barriers. In samples with barrier formation, CD8^+^ T and NK cells were restricted to the fibroblast-rich regions near the tumor, whereas in samples without barrier formation, they were distributed within the tumor region (Figure 2F). We also found that immune effector molecules were less abundant in the tumor region, thereby generating a CAF barrier (Figure S3H). By integrating ST and SM data, we found that the barrier region was significantly enriched with lipid metabolites (Figure S4A and B). Consistent with these findings, ST data revealed significant enrichment of the lipid synthesis-related genes *ACACA* [24], *ACLY* [25], and *FASN* [26] in the barrier region (Figure S4C). Some studies have shown that lipids have cytotoxic effects on immune cells [27–29]. These findings suggest that the enrichment of lipid metabolites in barrier regions may be a significant factor contributing to the decrease in the infiltration of immune cells. These results highlight multiple cell types involved in immunosuppressive mechanisms, including (1) the recruitment of Tregs, (2) B cells that exhibit Bregs characteristics, (3) exhausted T cells, (4) insufficient recruitment of FGFBP2^+^ NK cells, and (5) the formation of a spatial barrier niche with a decrease in the number of effector immune cells and an increase in the enrichment of lipids.

### Two recurring tumor epithelial subpopulations

**Figure 3:**
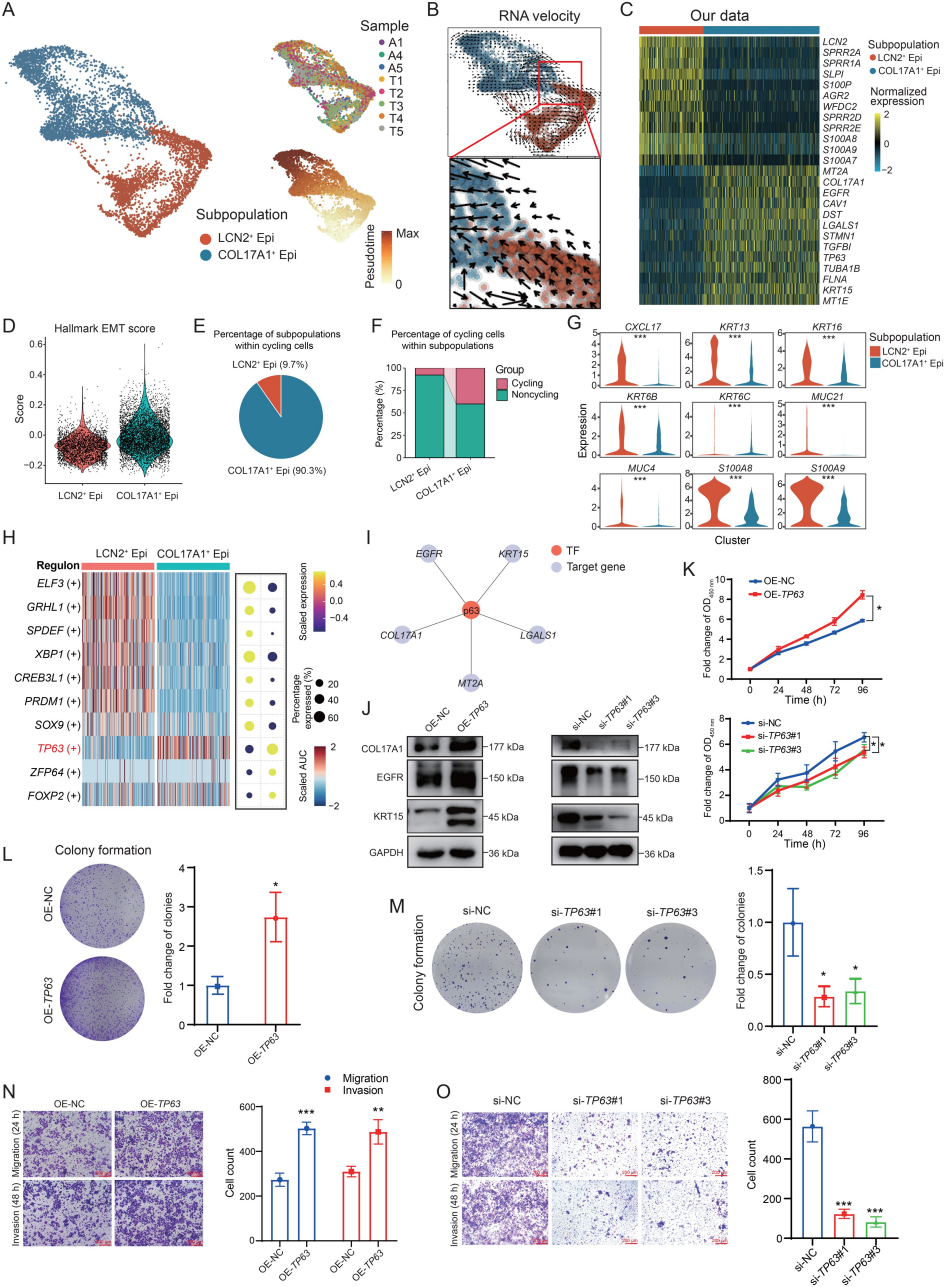
Identification of two recurrent subpopulations in epithelial cells **A**. UMAP plots showing epithelial cells labeled by two subpopulations, samples, and pseudotime, respectively. **B**. Single-cell trajectory inference of epithelial cells using scVelo. **C**. Heatmap showing the top 13 highly expressed genes in each epithelial subset. **D**. EMT pathway activity scores in epithelial subpopulations. **E**. Percentage of epithelial subpopulations within cycling cells. **F**. Bar plots showing the percentage of cycling cells within epithelial subpopulations. **G**. Violin plots showing the expression of epidermis maturation-related genes, including *KRT16*, *KRT13*, *KRT6B*, *KRT6C*, *MUC21*, *MUC4*, *CXCL17*, *S100A9*, and *S100A8* across epithelial cell subpopulations. **H**. Heatmap and dot plots showing the specific TF regulon AUC scores and mRNA expression in epithelial subpopulations. **I**. Regulatory network of the TF gene *TP63* and its downstream target genes. **J**. WB analysis showing the changes in protein expression levels of KRT15, COL17A1, and EGFR in KYSE-150 cells following *TP63* overexpression or knockdown. **K**. Cell viability was measured at 0, 24, 48, 72, and 96 h after *TP63* overexpression or knockdown in KYSE-150 cells using a CCK-8 assay (*n* = 3). **L**. and **M**. Quantification of colony formation efficiency in KYSE-150 cells following *TP63* overexpression (L) or knockdown (M) (*n* = 3). **N**. and **O**. Representative migration and invasion images of KYSE-150 cells in Transwell chambers at 24 and 48 h after *TP63* overexpression (N) or knockdown (O) (*n* = 3). Scale bar = 200 μm. *, *P* < 0.05; **, *P* < 0.01; ***, *P* < 0.001. Statistical significance was calculated by Student’s *t*-test, mean ± standard deviation (SD). EMT, epithelial-mesenchymal transition; TF, transcription factor; AUC, area under the curve; CCK-8, Cell Counting Kit-8; WB, Western blotting; OE, overexpression; NC, negative control.

After unsupervised clustering, all epithelial cells were divided into nine subgroups (Figure S5A). Further copy number variation (CNV) analysis revealed that subgroups 1, 7, and 8 had the lowest CNV scores, suggesting that these cells were nonmalignant (Figure S5B and C). To eliminate the effect of nonmalignant cells, we excluded these cells from the heterogeneity analysis. After reclustering all malignant epithelial cells, two epithelial clusters were identified, each expressing representative genes, including LCN2^*+*^ cells (marked by the genes *LCN2*, *SLPI*, and *S100P*) and COL17A1^+^ cells (marked by the genes *COL17A1*, *EGFR*, and *KRT15*) (**Figure 3**A, Figure S5D). Epithelial cells from the external dataset could also be classified into two subpopulations based on the top 13 cell type-specific genes for each epithelial subpopulation (Figure 3C, Figure S5E). Functional analysis revealed that LCN2^*+*^ epithelial cells were enriched in pathways related to the maturation of squamous epithelial cells, such as epidermis differentiation, development, and keratinization, whereas COL17A1^*+*^ epithelial cells were enriched in extracellular matrix (ECM) formation (Figure S5F). COL17A1^+^ epithelial cells preferentially occurred in tumor tissues, implying increased malignancy (Figure S5G). COL17A1^+^ epithelial cells exhibited higher epithelial-mesenchymal transition (EMT) scores (Figure 3D) and a greater proportion of proliferating cells (Figure 3E and F); these cells were associated with poorer patient prognosis (Figure S5H). These cells also exhibited lower expression levels of squamous epithelial differentiation-related genes, such as *KRT16*, *KRT13*, *KRT6B*, *KRT6C*, *MUC21*, *MUC4*, *CXCL17*, *S100A8*, and *S100A9* [30,31], further indicating that COL17A1^+^ epithelial cells were more malignant and in a dedifferentiated state (Figure 3G). Subsequent cell trajectory analysis confirmed the dedifferentiated state of COL17A1^+^ epithelial cells (Figure 3A and B). These results indicate that COL17A1^+^ epithelial cells may be more aggressive and malignant and that LCN2^+^ cells are tumor progenitor cells.

Transcription factors (TFs) are key determinants of cell fate. Therefore, we performed single-cell regulon network inference using the SCENIC software [32] (Figure 3H). We found that the TF gene *TP63* was most highly expressed in COL17A1^+^ epithelial cells, and its target genes, including *COL17A1*, *EGFR*, *MT2A*, *KRT15*, and *LGALS1*, were characteristic genes of COL17A1^+^ epithelial cells (Figure 3C and I). These findings suggest that *TP63* may be closely related to the lineage differentiation of COL17A1^+^ epithelial cells. To test this hypothesis, we conducted *in vitro* experiments in ESCC cell lines. The results revealed that the overexpression of *TP63* promoted the expression of the mRNAs of these characteristic genes, whereas knockdown of *TP63* reduced their mRNA levels (Figure S6A–D). We also found that the overexpression of *TP63* increased the protein levels of COL17A1, EGFR, and KRT15 (Figure 3J). These findings suggest that *TP63* is a crucial determinant of the fate of COL17A1^+^ epithelial cells.

To validate the function of *TP63*, we examined changes in cell function after overexpression or knockdown of *TP63* in ESCC cells. The results indicated that overexpression of *TP63* significantly increased tumor cell viability, clonogenicity, as well as migration and invasion capabilities, whereas knockdown of *TP63* had the opposite effect (Figure 3K–O). In summary, we identified two epithelial subpopulations in ESCC tissues: COL17A1^+^ epithelial cells exhibit increased malignancy, and the TF gene *TP63* plays a key role in maintaining the malignant phenotype.

### Spatial heterogeneity of epithelial cells

**Figure 4:**
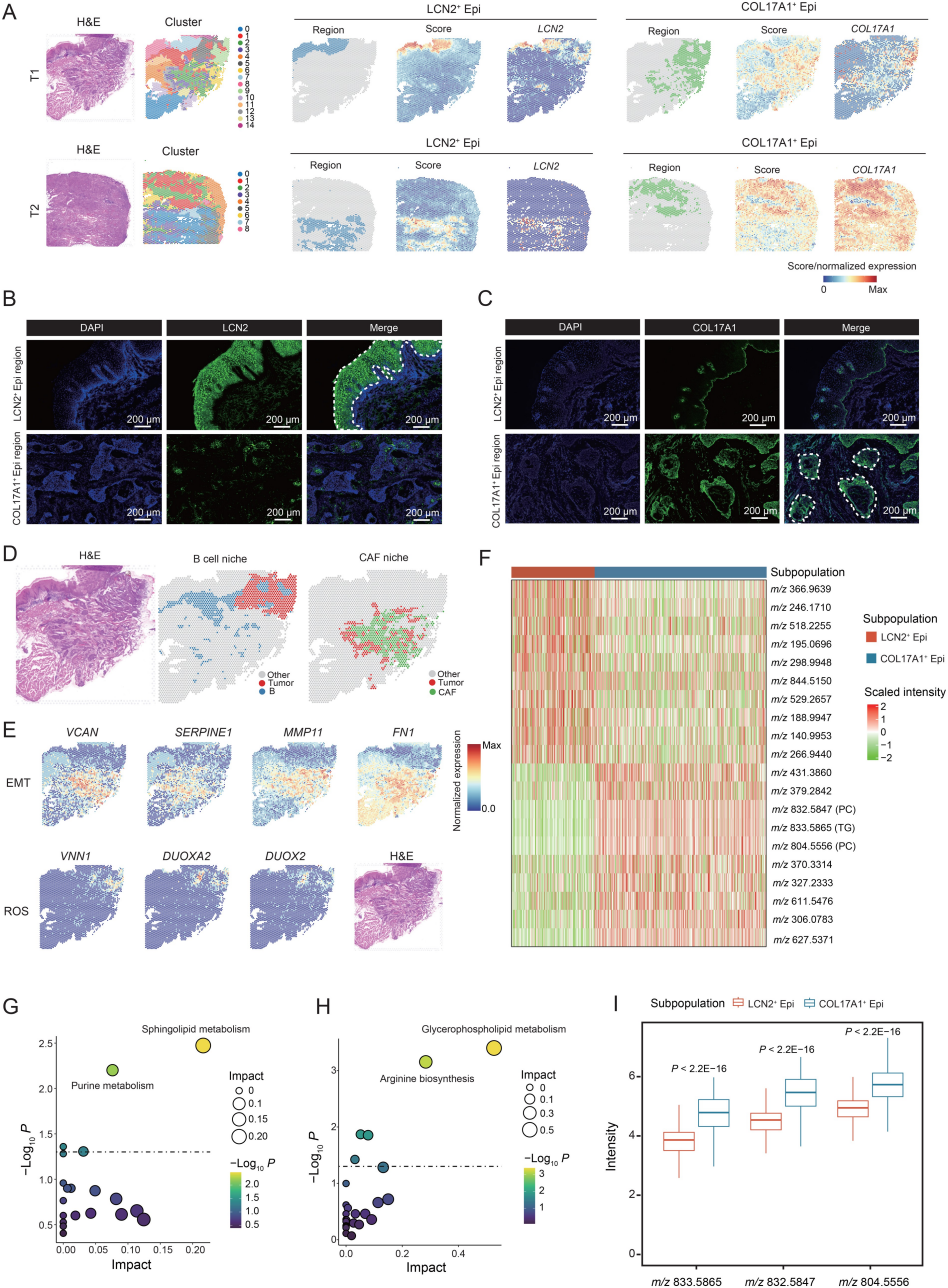
Spatial characteristics of epithelial subpopulations **A**. Left: H&E-stained tissue sections, followed by ST spot unbiased clustering. Right: spatial feature plots showing the epithelial subpopulation region, epithelial scores, and the expression of marker genes, including *LCN2* and *COL17A1*, in tissue sections for tumor samples 1 and 2. **B**. and **C**. IF staining of LCN2 (B) and COL17A1 (C) in LCN2^+^ and COL17A1^+^ epithelial regions from tumor sample 1. Scale bar = 200 μm. **D**. Left: H&E staining of tissue sections of tumor sample 1. Right: spatial spots of COL17A1^+^ epithelial cells in B cell or CAF niches. **E**. Spatial feature plots of selected genes, including *VCAN*, *SERPINE1*, *MMP11*, *FN1*, *VNN1*, *DUOXA2*, and *DUOX2* in tumor sample 1. **F**. Heatmap of the top 10 differentially enriched metabolites for each epithelial cell subpopulation. **G**. Dot plots showing the enriched pathways of upregulated metabolites in LCN2^+^ epithelial cells. **H**. Dot plots showing the enriched pathways of upregulated metabolites in COL17A1^+^ epithelial cells. **I**. Box plots showing the enrichment levels of PC (*m/z* 832.5847, *m/z* 804.5556) and TG (*m/z* 833.5865) in epithelial cell subpopulations. *P* < 0.05 (Wilcoxon test) is considered a statistically significant difference. IF, immunofluorescence; PC, phosphocholine; TG, triglyceride; ROS, reactive oxygen species.

To delineate the spatial heterogeneity of epithelial cells, two epithelial subpopulations were mapped to ST data. This analysis revealed that distinct epithelial subpopulations had a regionally restricted distribution tendency (**Figure 4**A). The marker genes *LCN2* and *COL17A1* also showed spatial patterns similar to those of the deconvolution scores (Figure 4A). Immunofluorescence experiments confirmed these distinct spatial expression features (Figure 4B and C). We also found that the spatial microenvironment was correlated with transcriptomic alterations in COL17A1^+^ epithelial cells. For example, the distribution patterns of COL17A1^+^ epithelial cells included two distinct niches: those adjacent to B cells and those intertwined with fibroblasts, as described in the tumor sample 1 (Figure 4D). In COL17A1^+^ epithelial cells from the fibroblast niche, the upregulated genes were enriched in the EMT-related pathway, whereas those from the B cell niche were associated with the reactive oxygen species (ROS) pathway, reflecting the plasticity of tumor cell states driven by spatial organization (Figure 4E, Figure S7A). The results of the CNV analysis revealed that spatial factors could drive tumors to form distinct subclonal populations (Figure S7B and C), suggesting that spatial variables are correlated with transcriptomic plasticity and affect genomic variation in tumor cells.

To evaluate the metabolic heterogeneity of epithelial cells, we used a deconvolution method to determine the abundance and spatial distribution of the two epithelial cell types (Figure S7D and E). We then integrated SM data to identify their metabolic profiles. Differential analysis revealed 155 significantly increased metabolites in the COL17A1^+^ tumor region and 400 significantly increased metabolites in the LCN2^*+*^ tumor region (Figure S7F). The top 10 differentially enriched metabolites in each tumor region are shown in Figure 4F. Further functional enrichment analysis revealed that LCN2^+^ epithelial cells were enriched in sphingolipid metabolism and purine metabolism, whereas COL17A1^+^ epithelial cells were enriched in arginine biosynthesis and glycerophospholipid metabolism (Figure 4G and H). The procarcinogenic metabolites triglycerides (TGs) and phosphocholines (PCs) significantly accumulated in the COL17A1^+^ tumor region, reflecting greater lipid accumulation in COL17A1^+^ epithelial cells [33,34] (Figure 4I). We found that the mRNA levels of diacylglycerol-acyltransferase 1 (*DGAT1*) and lysophosphatidylcholine acyltransferase 1 (*LPCAT1*), which are involved in the synthesis of TG and PC, were also high in COL17A1^+^ epithelial cells, as indicated by the scRNA-seq and ST data (Figure S7G–I). Overall, we found that the two types of epithelial cells occupied distinct spatial niches and that spatial factors were closely associated with the transcriptomic and genomic plasticity of tumors. We also determined their different metabolomic characteristics.

### POSTN^+^ fibroblasts are associated with tumor progression

**Figure 5:**
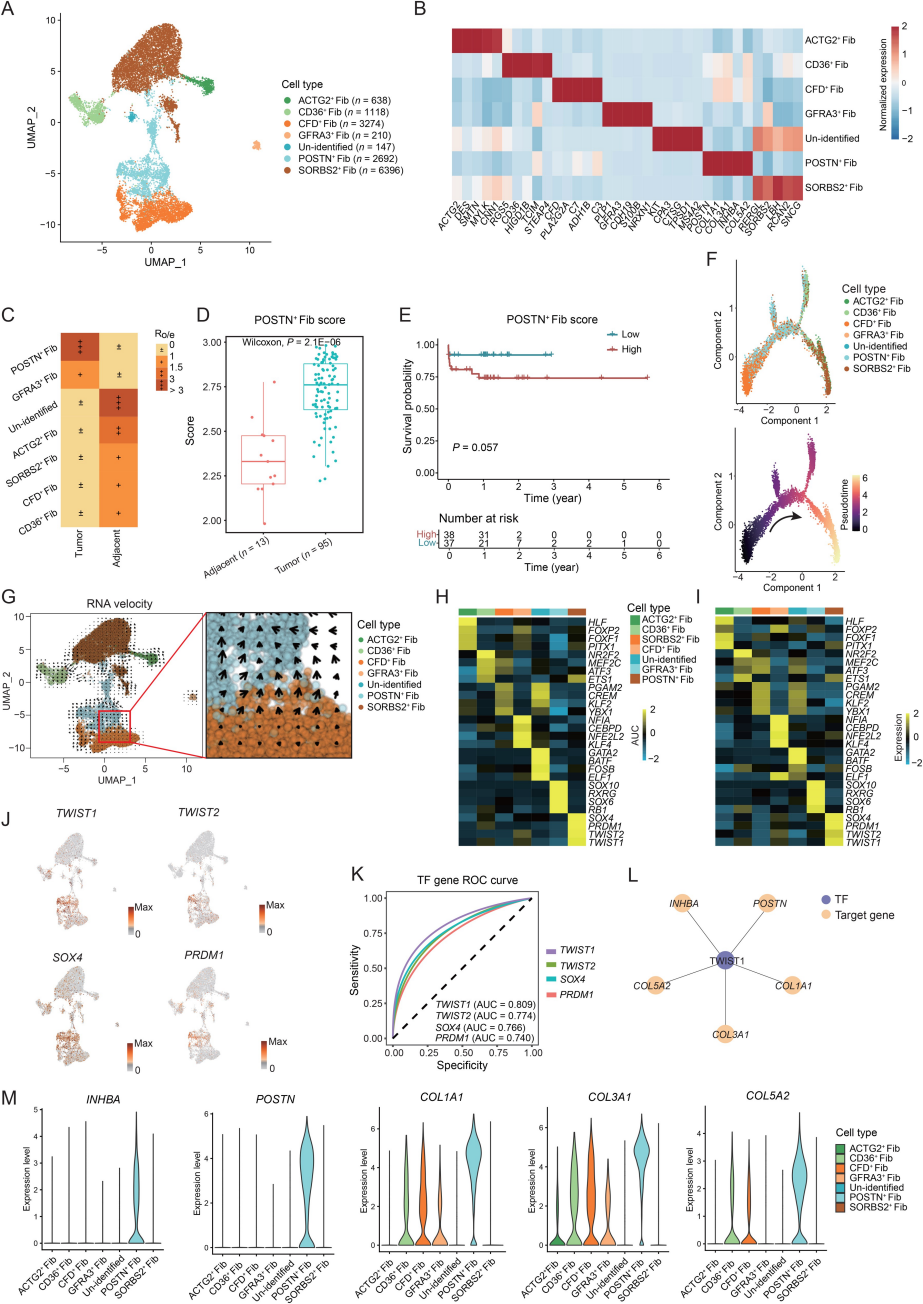
Analysis of fibroblast heterogeneity **A**. UMAP plots showing total fibroblasts labeled by seven fibroblast subpopulations. **B**. Heatmap showing the mRNA expression of the top 5 marker genes for seven fibroblast subpopulations. **C**. Comparison of the tissue preference distribution among seven fibroblast subpopulations in paired samples (*n* = 3). The R_o/e_ value indicates tissue preference, with R_o/e_ > 1 suggesting enrichment in tumor tissues and R_o/e_ < 1 indicating a preference for adjacent non-tumor tissues. **D**. Box plots showing the enrichment scores of POSTN^+^ fibroblasts between tumor (*n* = 95) and normal tissues (*n* = 13) in the ESCC dataset from The Cancer Genome Atlas (TCGA) database. *P* < 0.05 (Wilcoxon test) is considered a statistically significant difference. **E**. Survival curve of POSTN^+^ fibroblast scores in 75 ESCC samples. *P* < 0.05 (two-sided log-rank test) is considered a statistically significant difference. **F**. UMAP plots of cell trajectory analysis labeled by fibroblast subpopulations and pseudotime. **G**. Trajectory analysis of fibroblasts using scVelo. Heatmap showing the AUC scores (**H**) and mRNA expression (**I**) of the top 4 TF regulons for each fibroblast subpopulation. **J**. Feature plots of TF genes, including *TWIST1*, *SOX4*, *TWIST2*, and *PRDM1* in fibroblasts. **K**. ROC curve analysis showing the specificity of TFs *TWIST1*, *TWIST2*, *SOX4*, and *PRDM1* in POSTN^+^ fibroblasts. **L**. Regulatory network of TF gene *TWIST1* and its selected target genes. **M**. Violin plots showing the mRNA expression of *INHBA*, *POSTN*, *COL1A1*, *COL3A1*, and *COL5A2* among fibroblast subpopulations. ROC, receiver operating characteristic.

To investigate the heterogeneity and functionality of fibroblasts in ESCC, we first eliminated batch effects across samples and conducted a clustering analysis (Figure S8A and B). The results indicated that based on the expression levels of marker genes, fibroblasts could be categorized into seven subpopulations: ACTG2^+^ fibroblasts, CD36^+^ fibroblasts, SORBS2^+^ fibroblasts, CFD^+^ fibroblasts, GFRA3^+^ fibroblasts, POSTN^+^ fibroblasts, and unidentified fibroblasts (**Figure 5**A and B). Cell proportion analysis revealed that the abundance of fibroblast subgroups differed among tumor samples, reflecting the heterogeneity of the TME (Figure S8C). Functional enrichment analysis indicated that ACTG2^+^ fibroblasts were associated with oxidative phosphorylation and energy derivation by oxidation, CD36^+^ and SORBS2^+^ fibroblasts were associated with vascular processes in the circulatory system and muscle system processes, CFD^+^ fibroblasts were involved in immune regulatory functions, GFRA3^+^ fibroblasts were connected to axon development, and POSTN^+^ fibroblasts were related to the organization of extracellular structures (Figure S8D). We found that GFRA3^+^ and POSTN^+^ fibroblasts, especially POSTN^+^ fibroblasts, were preferentially distributed within tumor tissues, whereas other types of fibroblasts were predominantly located in adjacent non-tumor tissues, suggesting that they may participate in tumor progression (Figure 5C). Further large-scale analysis demonstrated that the enrichment of POSTN^+^ fibroblasts was greater in tumor tissues than in normal tissues and was linked to poor prognosis (Figure 5D and E).

We next performed cell trajectory analysis of these fibroblast subpopulations to determine the hierarchies of fibroblast differentiation. POSTN^+^ fibroblasts were positioned behind CFD^+^ fibroblasts in the inferred cell trajectory, suggesting that they probably originated from CFD^+^ fibroblasts (Figure 5F and G). Additionally, single-cell regulatory network inference was performed to identify subpopulation-specific TFs, which included the genes *TWIST1*, *TWIST2*, *SOX4*, and *PRDM1*, as TFs potentially controlling the fate of POSTN^+^ fibroblasts (Figure 5H). The mRNA expression levels of these TF genes were also greater in POSTN^+^ fibroblasts than in other cells (Figure 5I and J). Receiver operating characteristic (ROC) curve analysis revealed that *TWIST1* exhibited the highest specificity for POSTN^+^ fibroblasts (Figure 5K). *TWIST1* downstream genes, including *POSTN*, *INHBA*, *COL1A1*, *COL3A1*, and *COL5A2*, were identified as characteristic genes of POSTN^+^ fibroblasts (Figure 5L and M). Thus, we selected *TWIST1* for experimental validation. Overexpressing *TWIST1* in the BJ cells significantly increased the mRNA expression levels of these genes, whereas knocking down *TWIST1* produced an opposite effect (Figure S8E–H). These findings indicate that *TWIST1* is a key determinant of the fate of POSTN^+^ fibroblasts. To summarize, we have identified an important protumor fibroblast subgroup, POSTN+ fibroblasts, and found that *TWIST1* is a key TF regulating their fate.

### POSTN^+^ fibroblasts and COL17A1^+^ epithelial cells are the dominant cell types in the immune barrier region

**Figure 6:**
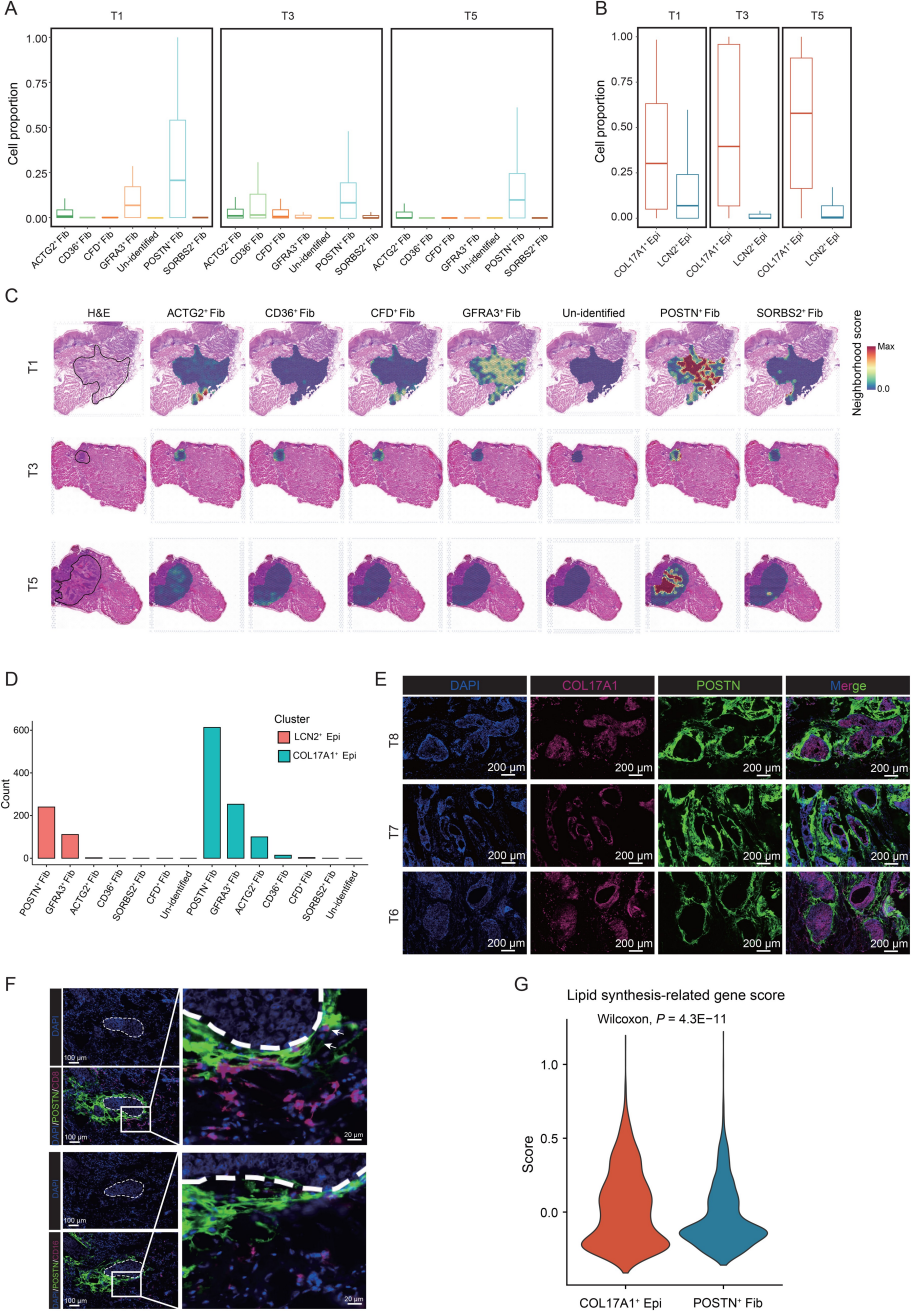
Identification of the components of the barrier niche **A**. Box plots showing the enrichment scores of seven fibroblast subpopulations in annotated CAF barrier regions across tumor samples 1, 3, and 5. **B**. Box plots showing the enrichment scores of two epithelial subpopulations in annotated CAF barrier regions across tumor samples 1, 3, and 5. **C**. Left: H&E staining with annotated CAF barrier regions (solid lines) in tumor samples 1, 3, and 5. Right: spatial feature plots showing the neighborhood scores of fibroblast subpopulations within spatial barrier niches. **D**. Bar plots showing the number of neighboring spots between epithelial and fibroblast subpopulations. **E**. Multi-color IF of COL17A1 and POSTN. Scale bar = 200 μm. **F**. Representative multi-color IF staining of POSTN, CD8, and CD16 in the immune barrier regions from tumor sample 1. POSTN represented fibroblasts, CD8 represented CD8^+^ T cells, and CD16 represented NK cells. Scale bar = 100 μm. **G**. Violin plots comparing the lipid synthesis-related gene signature (*ACACA*, *ACLY*, and *FASN*) scores between COL17A1^+^ epithelial cells and POSTN^+^ fibroblasts. *P* < 0.05 (Wilcoxon test) is considered a statistically significant difference.

We found that a CAF barrier was formed in ESCC (Figure 2F). To decipher how the structure was organized, we first extracted spots in the CAF barrier and calculated the enrichment levels of tumor cells and fibroblasts via spatial deconvolution in Seurat. We found that POSTN^+^ fibroblasts and COL17A1^+^ epithelial cells were the predominant cell types in the CAF barrier (**Figure 6**A and B). Cell neighborhood detection indicated that POSTN^+^ fibroblasts and COL17A1^+^ epithelial cells had the greatest number of colocalized spots in the barrier region, reflecting their spatial proximity (Figure 6C and D). To confirm our findings, the distribution patterns of POSTN and COL17A1 in the barrier region were assessed through immunofluorescence experiments. POSTN surrounded the tumor nests, forming a barrier structure, while COL17A1 was highly expressed in the tumor nests (Figure 6E). We also found that the marker proteins for CD8^+^ T cells or NK cells, CD8 and CD16, were predominantly distributed outside the POSTN^+^ barrier (Figure 6F). Moreover, SM data revealed that the immune barrier region was significantly enriched in lipid metabolites (Figure S4B). Therefore, we further investigated which cell type contributed to lipid enrichment in the barrier region by comparing the enrichment levels of lipid synthesis-related genes. Our results revealed that COL17A1^+^ epithelial cells had higher lipid synthesis-related gene signature scores, suggesting they were major contributors to lipid enrichment in the barrier region (Figure 6G). These results indicate that the immune barrier primarily consists of POSTN^+^ fibroblasts, which may shield immune surveillance in COL17A1^+^ epithelial cells. Additionally, COL17A1^+^ epithelial cells may further impair immune cell activity by enhancing lipid metabolism.

### POSTN^+^ fibroblast and COL17A1^+^ epithelial cell interactions contribute to tumor progression and immune barrier formation

**Figure 7:**
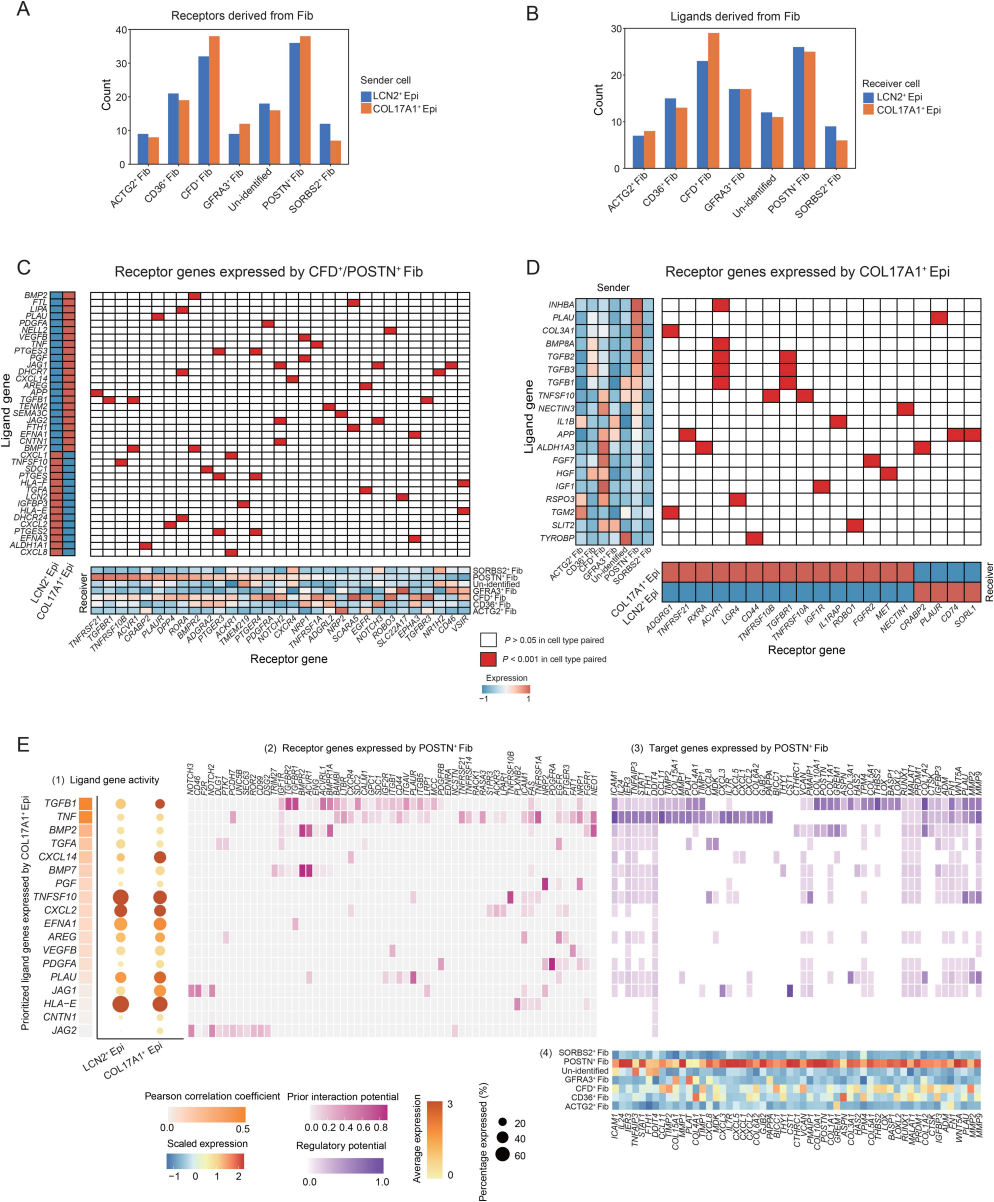
L–R interaction between epithelial cells and fibroblasts **A**. and **B**. Bar plots of significant L–R pairs (*P* < 0.001, permutation test) from epithelial cells to fibroblasts (A) or fibroblasts to epithelial cells (B) in scRNA-seq data. **C**. Left: scRNA-seq average expression of epithelial-derived ligands modulating POSTN^+^ or CFD^+^ fibroblasts. Bottom: scRNA-seq average expression of corresponding receptors in POSTN^+^/CFD^+^ fibroblasts. Middle: heatmap of significant L–R pairs from COL17A1^+^ epithelial cells to POSTN^+^ or CFD^+^ fibroblasts. **D**. Left: heatmap showing scRNA-seq average expression of POSTN^+^ fibroblast-derived ligands modulating COL17A1^+^ epithelial cells. Bottom: heatmap of scRNA-seq average expression of corresponding receptors in COL17A1^+^ epithelial cells. Middle: heatmap of significant L–R pairs from POSTN^+^ fibroblasts to COL17A1^+^ epithelial cells in scRNA-seq. **E**. (1) Dot plots showing the expression of top activity ligands in COL17A1^+^ epithelial cells from scRNA-seq; (2) Heatmap of prior interaction potential scores of ligand-matched receptors in POSTN^+^ fibroblasts; (3) Heatmap of regulatory potential scores of ligand-matched target genes in POSTN^+^ fibroblasts; and (4) Heatmap of scRNA-seq average expression of target genes in POSTN^+^ fibroblasts. L–R, ligand–receptor.

To investigate the mechanisms underlying the formation of this unique epithelial-fibroblast niche, we integrated scRNA-seq and ST data to resolve signaling communication between the two cell types. Based on the integrated ligand–receptor (L–R) pair pool from public datasets, we identified interactions between POSTN^+^ fibroblasts and COL17A1^+^ epithelial cells using CellPhoneDB software. We detected more L–R pairs between POSTN^+^ fibroblasts and COL17A1^+^ epithelial cells than between other cell-type pairs, suggesting a robust interaction between the two cell types (**Figure 7**A and B). Consistent with the spatial niches of POSTN^+^ fibroblasts and COL17A1^+^ epithelial cells, prominent COL17A1^+^ epithelial cell signaling to POSTN^+^ fibroblasts was mediated by several L–R pairs, such as TGFB1–TGFBR1 and BMP2–BMPR2 (Figure 7C, Figure S9A). In contrast, POSTN^+^ fibroblasts might modulate COL17A1^+^ epithelial cells through L–R pairs such as COL3A1–CD44, BMP8A–CRABP2, and PLAU–ADORA2B (Figure 7D, Figure S9B). CFD^+^ fibroblasts (a precursor of POSTN^+^ fibroblasts) also exhibited extensive communication with epithelial cells, such as FTH1/FTL–SCARA5, EFNA1–EPHA3, and JAG1–NOTCH2 (Figure 7C). Some studies have shown that these signaling pathways can increase the expression of TFs (TWIST1 and SOX4) that control the fate of POSTN^+^ fibroblasts [35,36], highlighting that epithelial cells may promote the development of CFD^+^ fibroblasts into POSTN^+^ fibroblasts. Moreover, we detected high expression of *JAG1* in COL17A1^+^ epithelial cells and *NOTCH2* in CFD^+^ fibroblasts. We also observed colocalization of *JAG1* and *NOTCH2* in the ST data, which confirmed that COL17A1^+^ epithelial cells mediated the differentiation of CFD^+^ fibroblasts via JAG1–NOTCH2 regulators (Figure S9A and C).

Fibroblasts serve as primary producers of ECM components and contribute to the formation of desmoplastic structures, which limit immune cell infiltration [37]. We found that POSTN^+^ fibroblast-specific genes were enriched in ECM-related pathways, which aligned with their role in the generation of desmoplastic structures (Figure S9B). CellPhoneDB and spatial proximity analysis revealed strong interactions between COL17A1^+^ epithelial cells and POSTN^+^ fibroblasts (Figure 7A and B). Therefore, we investigated whether COL17A1^+^ epithelial cells promoted the ECM remodeling ability of POSTN^+^ fibroblasts. The results of the NicheNet analysis revealed that COL17A1^+^ epithelial cells showed high ligand activity for TGFB1, TNF, BMP2, and TGFA (Figure 7E). These ligands interact with receptors on POSTN^+^ fibroblasts, inducing the expression of target genes encoding collagens or matrix metallopeptidases, such as *MMP1*, *MMP2, MMP9, COL11A1*, *COL15A1*, *COL4A1*, *COL6A2*, *COL5A1*, *COL10A1, COL1A1, COL1A2,* and *COL3A1,* in POSTN^+^ fibroblasts (Figure 7E). The results of the Gene Ontology (GO) enrichment analysis revealed that these target genes were enriched in terms of ECM organization and the collagen metabolic process, supporting our hypothesis (Figure S9D). In contrast, the top active TFs in POSTN^+^ fibroblasts, including HGF, PLAU, FGF7, INHBA, RSPO3, SLIT2, IL1B, IGF1, TGFB2, and TGFB3, modulated the target genes in COL17A1^+^ epithelial cells, affecting pathways related to tumor progression, including epithelial cell proliferation, stem cell differentiation, and apoptotic signaling (Figure S9E and F). These findings indicate that the regulatory network between POSTN^+^ fibroblasts and COL17A1^+^ epithelial cells synergistically facilitates tumor progression and the formation of desmoplastic structures.

### POSTN^+^ fibroblasts producing INHBA promote tumor progression via the ACVR1/SMAD2/TP63 axis

**Figure 8:**
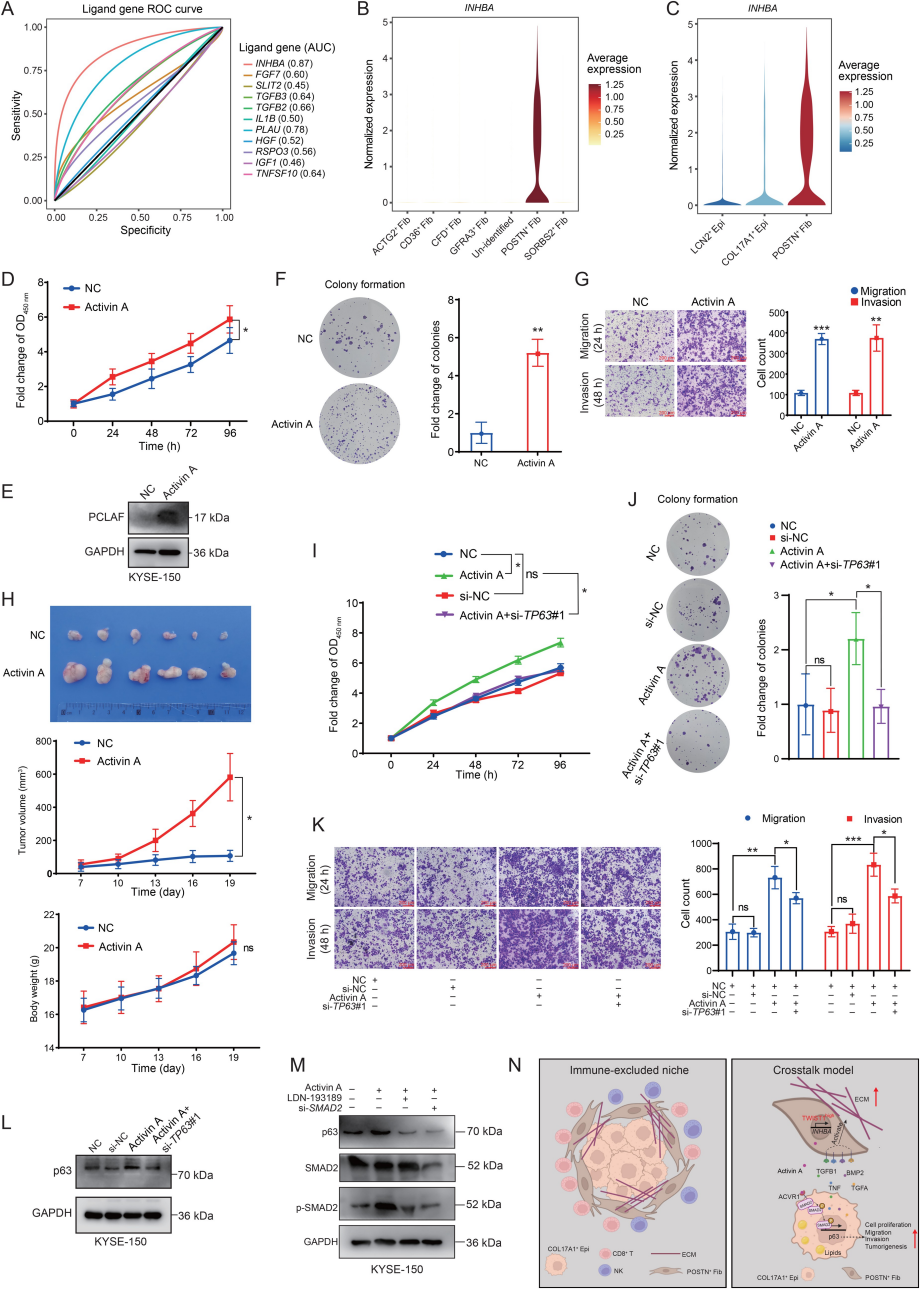
Validation of *INHBA* promoting tumor progression via the ACVR1/SMAD2/p63 axis **A**. ROC curve analysis showing the specificity of top active ligands derived from POSTN^+^ fibroblasts. **B**. Violin plots showing the mRNA expression levels of *INHBA* across seven fibroblast subpopulations. **C**. Violin plots showing the mRNA expression levels of *INHBA* between epithelial subpopulations and POSTN^+^ fibroblasts. **D**. Assessment of cell viability at 0, 24, 48, 72, and 96 h using a CCK-8 assay in KYSE-150 cells treated with activin A or the negative control. **E**. WB analysis of PCLAF in KYSE-150 cells after treatment with activin A or the negative control. **F**. Quantification of colony formation efficiency after treatment of KYSE-150 cells with activin A or the negative control. **G**. Transwell experiments examining the migration and invasion abilities of KYSE-150 cells at 24 and 48 h after treatment with activin A or the negative control (*n* = 3). Scale bar = 200 μm. **H**. Representative images, changes in volume and body weight of subcutaneous tumor xenografts treated with the negative control or activin A in BALB/c nude mice (*n* = 6). **I**. Measurement of cell viability at 0, 24, 48, 72, and 96 h using a CCK-8 assay after treatment of KYSE-150 cells with activin A or simultaneous treatment with si-*TP63* and activin A. **J**. Quantification of colony formation efficiency after treatment of KYSE-150 cells with activin A or simultaneous treatment with si-*TP63* and activin A (*n* = 3). **K**. Transwell experiments examining the migration and invasion abilities of KYSE-150 cells at 24 and 48 h after treatment with activin A or simultaneous treatment with si-*TP63* and Activin A (*n* = 3). Scale bar = 200 μm. **L**. WB analysis of p63 protein expression in KYSE-150 cells treated with activin A or transfected with si-*TP63*. **M**. WB analysis of p63, SMAD2, and p-SMAD2 protein expression in KYSE-150 cells treated with activin A and transfected with si-*SMAD2* or the ACVR1 inhibitor LDN-193189. **N**. Immune-excluded niche and crosstalk models of epithelial and fibroblast cells. Statistical significance was calculated by Student’s *t*-test (*, *P* <0.05; **, *P* <0.01; ***, *P* <0.001; ns, not significant).

Cell–cell communication analysis has revealed that POSTN^+^ fibroblasts play a key role in enhancing the malignant phenotype of tumor cells. To test this hypothesis, we conducted *in vivo* and *in vitro* experiments. Ligand-specific analysis revealed that among all possible ligand genes regulating tumor cells, *INHBA* had the highest expression specificity in POSTN^+^ fibroblasts (**Figure 8**A). Activin A (encoded by *INHBA*) is a secreted protein, and increasing activin A in tumor cells can promote tumor progression [38]. However, our data indicated that *INHBA* in the ESCC TME mainly originates from fibroblasts, suggesting that *INHBA* may be a key factor by which CAF promotes tumor progression (Figure 8B and C, Figure S10A). Additionally, *INHBA* was more highly expressed in tumor tissues than normal tissues and was positively correlated with the master TF gene of COL17A1^+^ epithelial cells, *TP63* (Figure S10B and C). Therefore, we selected *INHBA* for further experiments.

The results of the Cell Counting Kit-8 (CCK-8) assays revealed that treatment with the recombinant protein activin A significantly increased the viability of tumor cells (Figure 8D). Moreover, activin A also increased the expression of the cell proliferation marker PCLAF (Figure 8E). Clonogenic assays showed that activin A-treated tumor cells had enhanced colony-forming abilities (Figure 8F). Transwell assays also revealed that activin A treatment increased the migration and invasion capabilities of tumor cells, and xenograft experiments revealed that activin A enhanced the tumorigenic potential of tumor cells (Figure 8G and H). To determine whether activin A regulated the malignant phenotype of ESCC cells through *TP63*, we performed rescue experiments by simultaneously treating tumor cells with activin A and *TP63* siRNA (si-*TP63*), followed by monitoring changes in cell viability, colony formation, migration, and invasion. The results showed that knocking down *TP63* reversed the effects of activin A on promoting the malignant phenotype of tumor cells (Figure 8I–K). Moreover, simultaneous treatment of KYSE-150 cells with activin A and si-*TP63* reversed the ability of activin A to promote the expression of *TP63* (Figure 8L). Studies have shown that the INHBA–ACVR1 pathway activates the SMAD2 pathway, with SMAD2 acting as a co-TF to promote gene expression [39]. Therefore, we determined whether *INHBA* promotes *TP63* expression via the ACVR1/SMAD2 pathway. The results of gene manipulation experiments revealed that activin A promoted p63 expression and increased phosphorylated SMAD2 levels (Figure 8M). Treatment with si-*SMAD2* or an ACVR1 pathway inhibitor (LDN-193189) decreased p63 and phosphorylated SMAD2 levels, indicating that activin A promoted p63 expression through the ACVR1/SMAD2 pathway (Figure 8M). To summarize, *INHBA*, an essential oncogenic factor, is derived primarily from POSTN^+^ fibroblasts and promotes the malignant phenotype of tumor cells via the ACVR1/SMAD2/p63 axis in ESCC.

## Discussion

Although tumor immunotherapy has achieved remarkable curative outcomes in the treatment of various tumors, including ESCC [40], many patients who receive immunotherapy develop treatment resistance or failure due to the heterogeneity of tumor cells and the TME [41]. Therefore, the key molecular events underlying the TME need to be determined to identify predictive biomarkers and optimize immunotherapeutic strategies. We performed scRNA-seq to construct a single-cell transcriptomic atlas within ESCC and integrated multimodal spatial profiling to assess tumor transcriptomic and metabolic reprogramming and the TME architecture. In this study, the mechanisms underlying the formation of the immunosuppressive microenvironment were elucidated. The heterogeneity and interactions between tumor cells and fibroblasts were systematically evaluated, highlighting their key role in tumor progression.

The TME can be classified into three distinct types: the inflamed, the immune-excluded, and the immune-desert types [42]. In ESCC, we found that CD8^+^ T cells coexpressed immune effector and immunosuppressive molecules, reflecting the immune-exhausted state in the TME [43]. NK cells are important antitumor cells with promising therapeutic prospects [44]. We found that FGFBP2^+^ NK cells were in an activated state, as evidenced by their low expression of inhibitory molecules and favorable prognosis. However, the antitumor effect of FGFBP2^+^ NK cells may be limited by their low abundance in the TME. Therefore, our findings suggest that increasing the FGFBP2^+^ NK subpopulation in the TME may constitute an important antitumor strategy. Tregs play a crucial role in immune suppression [45], but their recruitment mechanism in the TME remains unclear. Our omics data revealed a pronounced chemotactic effect on Tregs in the TME via epithelial and stromal cells, including fibroblasts, mono-/macrophages, and cDCs. Along with Tregs, Bregs are involved in the inhibitory microenvironment [46]. However, the functions and molecular characteristics of Bregs remain unclear due to the limited number of relevant studies. In this study, B cells, particularly JCHAIN^+^ plasma cells, expressed the immunosuppressive genes *TGFB1* and *IGHG4* more widely in tumor tissues compared to adjacent non-tumor tissues. Moreover, some immunosuppressive genes (*VISR, LGALS9,* and *TNFRSF4*) were highly expressed in B cells. These results indicate that B cells, like Bregs, participate in the formation of a suppressive immune microenvironment.

Cancer cells exhibit differences in differentiation status, genomic variation, proliferation, and invasiveness among tumors and even within a tumor [47]. Hence, the precise identification of key conserved tumor cell subpopulations underlying tumor deterioration at the single-cell level may improve our understanding of tumorigenesis mechanisms and lead to the discovery of more universal therapeutic targets. We recapitulated two recurrent epithelial populations (LCN2^+^ and COL17A1^+^ epithelial cells) within ESCC by comprehensively integrating single-cell spatial multi-omic data and an external single-cell dataset. COL17A1^+^ epithelial cells are characterized by the activation of the EMT pathway, strong proliferation ability, and poor prognosis. We also identified key TFs determining the cell state of the two epithelial subpopulations. We found that the TF gene *TP63* was significantly activated in COL17A1^+^ epithelial cells and considerably increased the expression of COL17A1^+^ epithelial cell marker genes, such as *KRT15*, *COL17A1*, and *EGFR*, indicating that *TP63* played a key role in maintaining the state of COL17A1^+^ epithelial cells. Functional experiments revealed that *TP63* could significantly promote the malignant phenotype of esophageal cancer.

Some studies have shown that spatial information affects the transcriptomic reprogramming of tumors [48]. Consistent with this, the two epithelial subpopulations occupy distinct spatial niches, emphasizing the plasticity driven by spatial factors. Along with transcriptomic reprogramming, metabolic abnormalities are also hallmarks of tumor development [49]. SM was recently used to elucidate the metabolic heterogeneity of the TME [50]. However, given the lack of metabolic markers for the identification of cell types, determining the metabolic features of specific tumor or stromal cell subpopulations is challenging. In this study, we proposed an approach to integrate spatial transcriptomic and metabolic data to illustrate the metabolic heterogeneity of the interested subpopulations, using single-cell transcriptomics as a reference. Using our customized workflow, metabolic heterogeneity was determined between LCN2^+^ and COL17A1^+^ epithelial cells. COL17A1^+^ epithelial cells are characterized by the accumulation of TG and abnormal PC metabolism. This study provides a framework for investigating metabolic alterations in refined subpopulations.

CAFs play key roles in the solid tumors, influencing cancer progression [51]. Recent advances in scRNA-seq have provided a comprehensive understanding of the complexity and heterogeneity of CAF subsets in various types of cancer [52]. In this study, we systematically deciphered the transcriptomic heterogeneity of CAFs and identified a key protumorigenic fibroblast subpopulation, POSTN^*+*^ fibroblasts, characterized by ECM remodeling related to desmoplastic structures. We also identified the key TF TWIST1 in POSTN^+^ fibroblasts, which was closely associated with the function of POSTN^+^ fibroblasts. We identified an immune barrier composed of POSTN^+^ fibroblasts and COL17A1^+^ epithelial cells, with decreased infiltration of CD8^+^ T/NK cells and increased enrichment of lipids. Therefore, we propose an immune exclusion model in which POSTN^+^ fibroblasts encapsulate COL17A1^+^ epithelial cells, preventing immune cells from infiltrating the tumor core region. Additionally, the aberrant production of lipids by COL17A1^+^ epithelial cells further impairs the activity of immune cells (Figure 8N).

Given the unique niche formed by COL17A1^+^ epithelial cells and POSTN^+^ fibroblasts, we systematically mapped the mutual regulatory networks between the two cell types to elucidate the mechanisms underlying spatial niche formation. We found extensive cell communication between COL17A1^+^ epithelial cells and POSTN^+^ fibroblasts. We also found that ligands, such as TGFB1, TNF, BMP2, and TGFA, in COL17A1^+^ epithelial cells could enhance the matrix remodeling capabilities of POSTN^+^ fibroblasts, highlighting the role of COL17A1^+^ epithelial cells in the formation of this unique niche. In contrast, ligands such as HGF, PLAU, FGF7, INHBA, RSPO3, SLIT2, IL1B, IGF1, TGFB2, and TGFB3 in POSTN^+^ fibroblasts could promote the proliferation and differentiation of COL17A1^+^ epithelial cells. Our *in vitro* and *in vivo* experiments demonstrated that the ligand INHBA, derived from POSTN^+^ fibroblasts, could promote the expression of the key TF gene *TP63* in COL17A1^+^ epithelial cells via the ACVR1/SMAD2 pathway, suggesting that POSTN^+^ fibroblasts can facilitate the conversion of tumor cells into the COL17A1^+^ epithelial phenotype. Therefore, based on the above results, we proposed an interaction model between POSTN^+^ fibroblasts and COL17A1^+^ epithelial cells (Figure 8N).

## Conclusion

In summary, this study provides a comprehensive single-cell spatial multimodal resource for ESCC. We systematically illustrated the suppressive immune microenvironment in ESCC. We also investigated the heterogeneity of tumor cells and fibroblasts and elucidated the role of interactions between key tumor cells and fibroblast subpopulations in the formation of the immune barrier and promotion of tumor progression.

## Materials and methods

### Collection of clinical human patient samples

In this study, five ESCC tumor tissues and three patient-matched adjacent tissues were collected from the First Affiliated Hospital of Zhengzhou University. The patients were 65–69 years old. After detachment, the tissue was immediately transferred to the laboratory for further processing. The inclusion criteria for patients were a pathologist-confirmed diagnosis of ESCC and no other underlying diseases.

### Tissue dissociation and single-cell library construction

Fresh tissues were immediately preserved in a tissue preservation solution (Catalog No. LOT-4180011, Singleron, Nanjing, China) on ice. Tissues were dissociated following the manufacturer’s protocol. A scRNA-seq library was constructed using GEXSCOPE^®^ single-cell RNA library kits (Catalog No. LOT-4180011, Singleron) [53].

### ST

#### Tissue optimization

Tissue optimization was performed for ESCC tissues following the manufacturer’s recommendations. Briefly, eight optimal cutting temperature compound (OCT) embedded cryosections (10 μm thick) were placed on spatially optimized slides and treated with permeabilization enzymes (Catalog No. PN2000214, 10x Genomics, Pleasanton, CA) for different durations to determine the optimal penetration time. After determining the optimal conditions, the tissue sections were cut into 10-μm-thick sections and mounted onto spatial gene expression slides.

#### Fixation, staining, and imaging

Slides with sections were fixed in methanol for 20 min after incubation at 37°C for 1 min. Before staining, the slides were incubated in isopropyl alcohol (Catalog No. l9516, Sigma, St. Louis, MI) for 1 min. Then, the sections were incubated in Mayer’s hematoxylin (Catalog No. MHS16, Sigma) for 7 min and washed 15 times with ultrapure water. Next, the sections were incubated in bluing buffer (Catalog No. K3468, Dako, CA) for 1 min, and eosin (Catalog No. HT110216, Sigma) diluted 1:10 in Tris-base (0.45 M Tris, 0.5 M acetic acid, pH 6.0) was added for 1 min. After air-drying, the slides were imaged at 20× magnification using a 3DHISTECH platform (Catalog No. P1000, Budapest, Hungary).

#### Permeabilization

Slides with tissue sections were inserted into cassettes to establish individual reaction chambers for each section. For permeabilization, the sections were incubated at 37°C for 30 min with permeabilization enzymes. After incubation, the permeabilization enzyme was aspirated, and the wells were rinsed with 0.1× saline sodium citrate buffer.

### Spatial transcriptomic library preparation and sequencing

After permeabilization, cDNA synthesis and amplification (17 cycles) were performed using the Spatial Visium Reagent Kit (Catalog No. PN-1000189, 10x Genomics). The cDNA libraries were subsequently prepared using a spatial cDNA library kit (Catalog No. PN-1000196, 10x Genomics). The cDNA libraries were sequenced on the Illumina NovaSeq Xplus platform using an S4 Reagent Kit (Catalog No. 20084804, Illumina, San Diego).

### SM

#### Sample preparation

The OCT-embedded tissues were sliced at a thickness of 14 μm using a Leica CM3050S cryostat (Leica Microsystems GmbH, Wetzlar, Germany) at –20°C. The sections were subsequently mounted on electrically conductive slides coated with indium tin oxide (ITO) and then dried in a vacuum desiccator for 30 min.

#### Matrix coating and mass spectrometry imaging

Dehydrated samples were treated with an HTX TM sprayer (Bruker Daltonics, Bremen, Germany), and 2,5-dihydroxybenzoic acid (15 mg/ml) mixed in 90% acetonitrile was applied. Next, a prototype Bruker timsTOF flex mass spectrometry imaging system (Bruker Daltonics) equipped with a 10 kHz smartbeam 3D laser was used for MALDI timsTOF MSI analysis. The laser power was consistently maintained at 80% throughout the process. The positive-ion mode was selected to obtain mass spectra over a mass range of 50–1300 Da. The tissue images were resolved at a spatial resolution of 50 μm, with each spectrum compiled from 400 laser shots. Normalization was performed using the root mean square method on the MALDI mass spectra, and the signal intensity displayed in each image represented the normalized values. To confirm the structural identification of the detected metabolites, MS/MS fragmentation was conducted using the timsTOF flex MS system in the MS/MS mode.

### Immunofluorescence assay

For immunofluorescence staining, the samples were deparaffinized with xylene, rehydrated via an alcohol gradient, and washed with distilled water. Antigen retrieval was performed at 95°C for 30 min using sodium citrate buffer (0.01 M, pH 6.0). Then, the samples were sequentially blocked for 25 min and 30 min at 25°C in 3% hydrogen peroxide solution and bovine serum albumin (BSA, ST023, Beyotime, Shanghai, China) buffer (0.3% Triton X-100, 1% BSA) and then rinsed three times (5 min each) with phosphate-buffered saline (PBS). The samples were subsequently incubated for 10 h at 4°C with primary antibodies and then washed three times with PBS (5 min each). The samples were incubated for 50 min with horseradish peroxidase (c)-labeled secondary antibody (Catalog No. G1267-200T, Servicebio, Wuhan, China), followed by washing three times with PBS (5 min each). The samples were treated for 10 min with tyramide (Catalog No. G1231, Servicebio) and then washed three times with tris-buffered saline-tween (5 min each). Finally, the cell nuclei were stained with DAPI (Catalog No. G1012, Servicebio) for visualization, and fluorescence images were acquired. The primary antibodies used included rabbit anti-CD16 (1:3000, Catalog No. GB113963, Servicebio), mouse anti-CD8α (1:5000, Catalog No. GB12068, Servicebio), rabbit anti-Periostin (1:500, Catalog No. ab215199, Abcam, Waltham, MA), rabbit anti-COL17A1 (1:100, Catalog No. ab184996, Abcam), and rabbit anti-Lipocalin-2 (1:3000, Catalog No. 44058, CST, Danvers, MA) antibodies. The secondary antibodies used included goat HRP-labeled anti-IgG (1:500, Catalog Nos. GB23301 and GB23303, Servicebio). Goat anti-rabbit IgG (1:2000, Catalog No. A-11008, Thermo Fisher Scientific, Waltham, MA) was used. Tyramide included iF488-tyramide (1:500, Catalog No. G1231, Servicebio), iF555-tyramide (1:500, Catalog No. G1233, Servicebio).

### Cell culture

The KYSE-150 and BJ (human fibroblast) cell lines were purchased from the Cell Bank of the Chinese Academy of Sciences (Shanghai, China). KYSE-150 cells were cultured in RPMI-1640 medium (Catalog No. 12633020, Gibco, Grand Island, NY), while BJ cells were cultured in DMEM (Catalog No. 11965092, Gibco), both supplemented with 10% fetal bovine serum (FBS) (Catalog No. A5669701, Gibco). The cells were incubated at 37°C in a humidified atmosphere with 5% CO_2_.

### Cell proliferation, colony formation, and cell migration and invasion assays

Cell proliferation was assessed at predetermined intervals using CCK-8 (Catalog No. K1018, APExBIO, Houston, TX), following the manufacturer’s instructions. For the colony formation assay, cells were seeded in six-well plates at a density of 1000 cells per well in RPMI-1640 medium supplemented with 10% FBS. The cells were incubated at 37°C in a humidified atmosphere containing 5% CO_2_ for 14 days. The colonies were subsequently fixed with 4% paraformaldehyde, stained with crystal violet, and the visible colonies were counted. For the migration assay, cells (5 × 10^4^ cells per well) were suspended in 200 μl of FBS-free RPMI-1640 medium and added to a Transwell insert (Catalog No. 3450, Corning). The lower compartment of each well was filled with 500 μl of RPMI-1640 containing 10% FBS. For the invasion assay, Transwell chambers were precoated with 4% Matrigel (Catalog No. 354234, Corning, Tewksbury, MA) diluted in RPMI-1640 and incubated at 37°C for 1 h. The cells were then seeded in the upper chamber as described in the migration assay. After 48 or 72 h, the Transwell inserts were collected and stained with 0.5% crystal violet. Images of the stained cells on the lower surface of the inserts were captured, and the number of cells in three random fields was quantified under a microscope.

### Western blotting

Total proteins were extracted using RIPA lysis buffer (Catalog No. P0013B, Beyotime), and their concentrations were quantified by conducting the bicinchoninic acid (BCA) protein assay. The following primary antibodies were used in this study: anti-p63 (Catalog No. 12143-1-AP, Proteintech, Rosemont, IL), anti-COL17A1 (Catalog No. ab184996, Abcam), anti-Cytokeratin 15 (Catalog No. 10137-1-AP, Proteintech), anti-EGFR (Catalog No. 18986-1-AP, Proteintech), anti-GAPDH (Catalog No. 10494-1-AP, Proteintech), anti-SMAD2 (Catalog No. 12570-1-AP, Proteintech), anti-pSMAD2 (Catalog No. 280888, Abcam), anti-INHBA (Catalog No. 60352-1-lg, Proteintech), anti-TWIST1 (Catalog No. 25461-1-AP, Proteintech), and anti-Periostin (Catalog No. ab215199, Abcam).

### RNA interference

The siRNAs were prepared in Opti-MEM (Catalog No. 31985070, Gibco) and introduced into the cell lines using Lipofectamine 3000 (Catalog No. L3000150, Invitrogen, Carlsbad, CA) following the manufacturer’s protocol.

### *In vivo* tumorigenesis and metastasis assays

Null/null athymic BALB/c female mice (5–6 weeks old) were purchased from Beijing Vital River Laboratory Animal Technology Co., Ltd. (Beijing, China), and all procedures were performed according to the Animal Research Reporting of *In Vivo* Experiments (ARRIVE) guidelines. For xenograft implantation, KYSE-150 cells (5 × 10^6^ cells per mouse) were suspended in PBS and subcutaneously injected into the right flank of each mouse. After rearing for three days, the mice were administered 4 mg/kg recombinant activin A (Catalog No. P08476, Thermo Fisher Scientific) in 0.9% NaCl via intraperitoneal (i.p.) injection every two days. Tumor volumes were measured every 2 days using calipers, with volumes calculated using the formula V = (L × W^2^)/2, where L represents the longest diameter and W the shortest perpendicular diameter. After feeding for 20 days, the mice were euthanized, and the tumors were harvested for further analysis.

### Quantitative real-time polymerase chain reaction

Total RNA was isolated using TRIzol reagent (Catalog No. 15596026, Thermo Fisher Scientific) and subsequently reverse-transcribed into cDNA using the PrimeScript RT Reagent Kit with gDNA Eraser (Catalog No. RR047Q, Takara, San Jose, CA). Quantitative real-time polymerase chain reaction (qPCR) was conducted using TB Green^®^ Premix Ex Taq™ II (Tli RNaseH Plus) (Catalog No. RR420Q, Takara) following the manufacturer’s instructions. Next, 18S rRNA was used as an internal control, and gene expression levels were quantified using the 2^–ΔΔCt^ method.

### Construction of stably overexpressing cell lines

The *TP63* and *TWIST1* lentiviruses were constructed by GENECHEM (Shanghai, China). Then, *TP63* and *TWIST1* lentiviruses were transfected into KYSE-150 and BJ cells using HitransG P solution (REVG005, GENECHEM) for 24 h. Positive cells were selected with puromycin (2 µg/ml, Catalog No. P8230, Solarbio) and subsequently verified by Western blotting and qPCR assays.

### Raw sequence data processing

Human reference genome data were obtained from the 10x Genomics website (https://cf.10xgenomics.com/supp/cell-exp/refdata-gex-GRCh38-2020-A.tar.gz). CellRanger (10x Genomics) and Spaceranger (10x Genomics) were used to preprocess raw sequencing data to generate UMI counts for scRNA-seq and spatial transcriptomics data, respectively.

### Processing of scRNA-seq matrix data

Gene expression data were loaded into the R platform for analysis using the Seurat software [54]. Low-quality cells with fewer than 200 detected genes and more than 20% mitochondrial genes were removed before further analysis. Genes whose expression was under three cells were filtered out. The gene-barcode count matrices were normalized using the “SCTransform” function from Seurat. To decrease dimensionality, the top 3000 genes with high variability were identified using the “FindVariableGenes” function. Principal component analysis (PCA) was performed for dimensionality reduction. Then, all matrices were integrated into a combined dataset, and the batch effects from the samples were removed using the robust PCA (RPCA) algorithm. UMAP analysis was performed using the “RunUMAP” function with the top 30 principal components (PCs) as inputs. Cluster analysis for total cells was performed using the Louvain algorithm (resolution = 0.5).

### ST clustering analysis

Spatial gene expression and image data were analyzed using the R package Seurat. Spots not covered by the tissue section were removed from the downstream analysis. The “SCTransform” function was used to normalize the raw spot-barcode matrices. Dimensionality reduction was performed using the PCA algorithm for each spatial sample. Clustering analysis was also performed using the Louvain algorithm (resolution = 0.5) based on UMAP plots. Spot clusters were visualized in UMAP plots or spatial spots over H&E images. Factor analysis was conducted to determine the abundance of distinct cell populations in each spot using the “FindTransferAnchors” and “TransferData” modules in the Seurat software. The “SpatialFeaturePlot” function in Seurat was used to visualize the enrichment of distinct cell types.

### CNV analysis

Using the Pyinfercnv software, CNV analysis was performed on epithelial cells. Briefly, the raw single-cell matrix was used as the input file, and 1000 immune cells were randomly selected as the reference. The standard pyinfercnv pipeline was run to calculate CNV values.

### Identification of differentially expressed genes

Cell-specific marker genes were determined using the “Findallmarker” function in the Seurat software for each cell population. For a given cell type, the genes expressed in more than 10% of the cells with log_2_ fold change > 0.25 were regarded as marker genes. Using the “Findmarker” function in the Seurat software, spatial region-restricted genes were identified by comparing each compartment of interest in the tissue sections. Genes with log_2_ fold change > 0.5 and adjusted *P* < 0.05 were regarded as statistically significant spatially variable genes.

### Cell pseudotime analyses

Two methods were used for cell trajectory inference. (1) The cell development trajectory was inferred using the R package Monocle2. First, the single-cell UMI count matrices were normalized using the “estimateSizeFactors” and “estimateDispersions” modules in Monocle2. Genes with a mean value > 0.1 were retained for dimensionality reduction and the generation of the cell trajectory. Finally, the cell pseudotime trajectory was established using the “DDRTree” method in Monocle2. (2) The scRNA-seq data were preprocessed to obtain spliced and unspliced RNA counts for each gene in each cell, followed by normalization and filtering of low-quality cells and genes. Using the velocyto R package, RNA velocity vectors were estimated by computing the ratio of spliced to unspliced mRNA for each gene, and these vectors were projected onto a low-dimensional embedding to visualize inferred cell trajectories.

### GO and pathway enrichment analysis

GO and pathway enrichment analyses were performed using the R package clusterProfiler [55]. Gene identifiers were mapped using the annotation Dbi R package org.Hs.eg.db. All expressed genes were included as backgrounds. GO items with *P* < 0.05 were considered to be statistically significant. The results were visualized using the ggplot2 R package.

### Cell interaction analysis

L–R pairs were scanned using the CellPhoneDB [56] tool. First, we computed the average mRNA expression of L–R pairs across distinct cell–cell pairs in single-cell sequencing data. Only genes expressed in more than 10% of cells per cell type were considered available ligands or receptors. Then, CellPhoneDB was run to determine the available L–R pairs for each cell–cell pair, with default parameters and normalized count matrices as inputs. For statistically significant L–R pairs, the threshold was set at an average expression level > 0.2 and *P* < 0.001.

### TF regulon analysis

The SENIC [32] tool was used to determine the active transcription regulon in cell types of interest. A total of 2025 unique TFs were obtained by integrating resources from four TF databases, including JASPAR (http://jaspar.genereg.net/) [57], AnimalTFDB (https://guolab.wchscu.cn/AnimalTFDB4/) [58], hTFtarget (https://guolab.wchscu.cn/hTFtarget/#!/) [59], and TRRUST (https://www.grnpedia.org/trrust/) [60]. The hg38 human reference genome was obtained from a website (https://resources.aertslab.org/cistarget/databases/homo_sapiens/hg38/refseq_r80/mc9nr/gene_based/). First, we calculated the correlation between TF-target pairs to build a co-expression module using the GENIE3 algorithm in normalized scRNA-seq data. The TF regulons were subsequently determined according to the hg38 human genome reference data. Finally, the R package AUCell was used to calculate area under the curve (AUC) scores representing the transcriptional activity of the TF regulons. For cell-type-specific TF regulons, we used the Shannon entropy algorithm to determine the top-specific TFs for each cell cluster based on the AUC scores.

### Cell preference distribution analyses

To evaluate the differences in the distributions of fibroblast or epithelial cell subpopulations in cancer cells and adjacent tissues, the R_o/e_ value was calculated to represent the degree of tissue preference distribution:


$$R_{o/e}=\frac{Observed}{Expected}$$

For a given cell subpopulation *i* and its corresponding tissue *j*, a 2 × 2 contingency table was constructed. This table included the number of cells *i* in tissue *j* and other tissues. The number of non-*i* cells in tissue *j* and in other tissues was also counted. A Fisher’s exact test was subsequently performed on the contingency table to obtain the R_o/e_ value and the corresponding *P* value. A R_o/e_ value > 1 indicates that cell subpopulation *i* is more likely to be distributed in tissue *j*, whereas a lower R_o/e_ value < 1 indicates that cell subpopulation *i* is less likely to be distributed in tissue *j*.

### Alignment of metabolic and ST data

To integrate spatial transcriptomic and metabolomic data, we developed an image-based integration method. Briefly, we employed an image registration pipeline involving affine alignment and a nonlinear transformation to spatially co-register transcriptomic and metabolomic images. We used the simple ITK framework (Python software was used for medical image analysis) for image preprocessing and registration (https://simpleitk.org/). First, the ST H&E and SM images were converted to grayscale images and resampled to the same size. Then, we aligned the centers of the two images and registered the images via an affine transformation. A mutual information algorithm was used to evaluate similarity. The optimizer was established using a gradient descent algorithm. Using this approach, we obtained the transformation parameters of the registration. Finally, the pixel coordinates of the ST and SM images were converted to the same coordinate system. Because of the distinct resolutions of the ST and SM data, it was necessary to integrate the ST spots and SM pixels. For a given ST spot ${S=s}_{1}^{k}$, *i* = 1, …*k*, with *n* surrounding metabolic pixels $M=m_{1}^{n}$, *i* = 1, …*n*. The distance between *S* and *M* is $d_{\left|S-M\right|}<100\;\mu m$. We defined the metabolic intensities of spot *S* in $M_{s}$ using the following formula:


$$M_{s}=\;\frac{\sum_{i=1}^{n}m}{n}$$

### Identification of differentially abundant metabolites

The “FindAllMarkers” function in Seurat was used to compute differentially abundant metabolites in the four spatial niches. To identify differentially abundant metabolites between LCN2^+^ and COL17A1^+^ epithelial cells, the proportion of epithelial cell subpopulations in each spot in the epithelial region was first calculated using the deconvolution algorithm in Seurat. Next, the average proportion of each epithelial cell subpopulation in each spatial cluster in the epithelial region was determined. Spatial Seurat clusters with high average proportions were classified as corresponding epithelial cell subpopulation spatial clusters. Finally, the “FindAllMarkers” function in Seurat was used to calculate the differentially abundant metabolites in epithelial cell subpopulations. Log_2_ fold change > 1 and *P* < 0.05 were considered statistically significant.

### Cellular neighborhood detection

The method used by Dalia et al. was employed to detect the neighborhood of a cell in spatial data [61]. We defined the “neighborhood score” as the fraction of surrounding spots containing that cell type, thereby directly measuring the cell type composition in adjacent spots. The neighborhood of a spot was defined as spots with a distance less than or equal to 1 (including the spot), resulting in sets of no more than 7 neighbors per spot. The neighborhood cell type fraction was calculated from the binarized cell type annotations of this set. Briefly, we first calculated the neighborhood score for each fibroblast subgroup in the barrier region. We subsequently used the deconvolution algorithm to calculate the proportion of LCN2^+^ and COL17A1^+^ epithelial cells in each spot of the barrier region. Finally, we counted the number of neighborhood spots, defined as the proportion of LCN2^+^ or COL17A1^+^ epithelial cells, and the neighborhood score of each fibroblast subgroup was greater than 0.1 to evaluate the cellular neighborhood.

### Analysis of TCGA expression and survival curves

The ESCC bulk transcriptomic data with clinical information, including 95 tumor samples and 13 normal samples, were downloaded from The Cancer Genome Atlas (TCGA) database (https://www.cancer.gov/ccg/research/genome-sequencing/tcga) using the tool TCGAbiolinks [62]. Read count matrices were normalized using the transcripts per million (TPM) method. The TPM data were subsequently used to detect the expression of genes of interest. For survival analysis, 75 tumor samples remained after filtering out samples without clinical information. Survival curves were drawn using the R package survival. A Kaplan–Meier survival curve was generated using the “survfit” function in survival software.

## Ethical statement

The animal experiments were approved by the Ethics Committee of Scientific Research and Clinical Trial of the First Affiliated Hospital of Zhengzhou University (Approval No. ZZU-LAC20240531). The patient sample collection was approved by the Ethics Committee of Scientific Research and Clinical Trial of the First Affiliated Hospital of Zhengzhou University (Approval No. 2022-KY-1544-001). The written informed consent was obtained from all participants. The prior reporting for open utilization of human genetic resources of this project has been filed with the National Health Commission (NHC) (Notice No. 2025BAT00991).

## Code availability

The raw code reported in this study has been deposited at GitHub (https://github.com/biosyy/single-cell-and-spatial-transcriptomic). The code has also been submitted to BioCode at the National Genomics Data Center (NGDC), China National Center for Bioinformation (CNCB) (BioCode: BT007955), which is publicly accessible at https://ngdc.cncb.ac.cn/biocode/tool/BT007955.

## Supplementary Material

qzaf095_Supplementary_Data

## Data Availability

The raw data reported in this study have been deposited in the Genome Sequence Archive for Human [63] at the NGDC, CNCB (GSA-Human: HRA007162; BioProject: PRJCA025123), and are publicly accessible at https://ngdc.cncb.ac.cn/gsa-human.

## CRediT author statement

**Yong Shi:** Writing – original draft, Validation, Methodology, Formal analysis, Resources. **Ke An:** Software, Formal analysis. **Yu Qi:** Resources, Investigation. **Xinhan Zhang:** Validation. **Yueqin Wang:** Validation, Investigation. **Xuran Zhang:** Resources, Data curation. **Shaoxuan Zhou:** Writing – review & editing. **Ouwen Li:** Writing – review & editing. **Yanan Song:** Writing – review & editing. **Jiayi Zhou:** Writing – review & editing. **Yue Du:** Writing – review & editing. **Mingyang Hou:** Writing – review & editing. **Yun-Gui Yang:** Conceptualization, Supervision. **Xin Tian:** Funding acquisition, Conceptualization, Supervision. All authors have read and approved the final manuscript.
