# Supplementary material for: A Single-cell and Spatially Resolved Cell Atlas of Human Esophageal Squamous Cell Carcinoma
Source: Genomics Proteomics Bioinformatics. 2025 Nov 4;24(1):qzaf095. doi: 10.1093/gpbjnl/qzaf095 (PMC13433246; doi:10.1093/gpbjnl/qzaf095)
Supplement: Supplementary file 1 [file qzaf095_supplementary_data.zip › qzaf095_Supplementary_Data/FigS4.pdf]

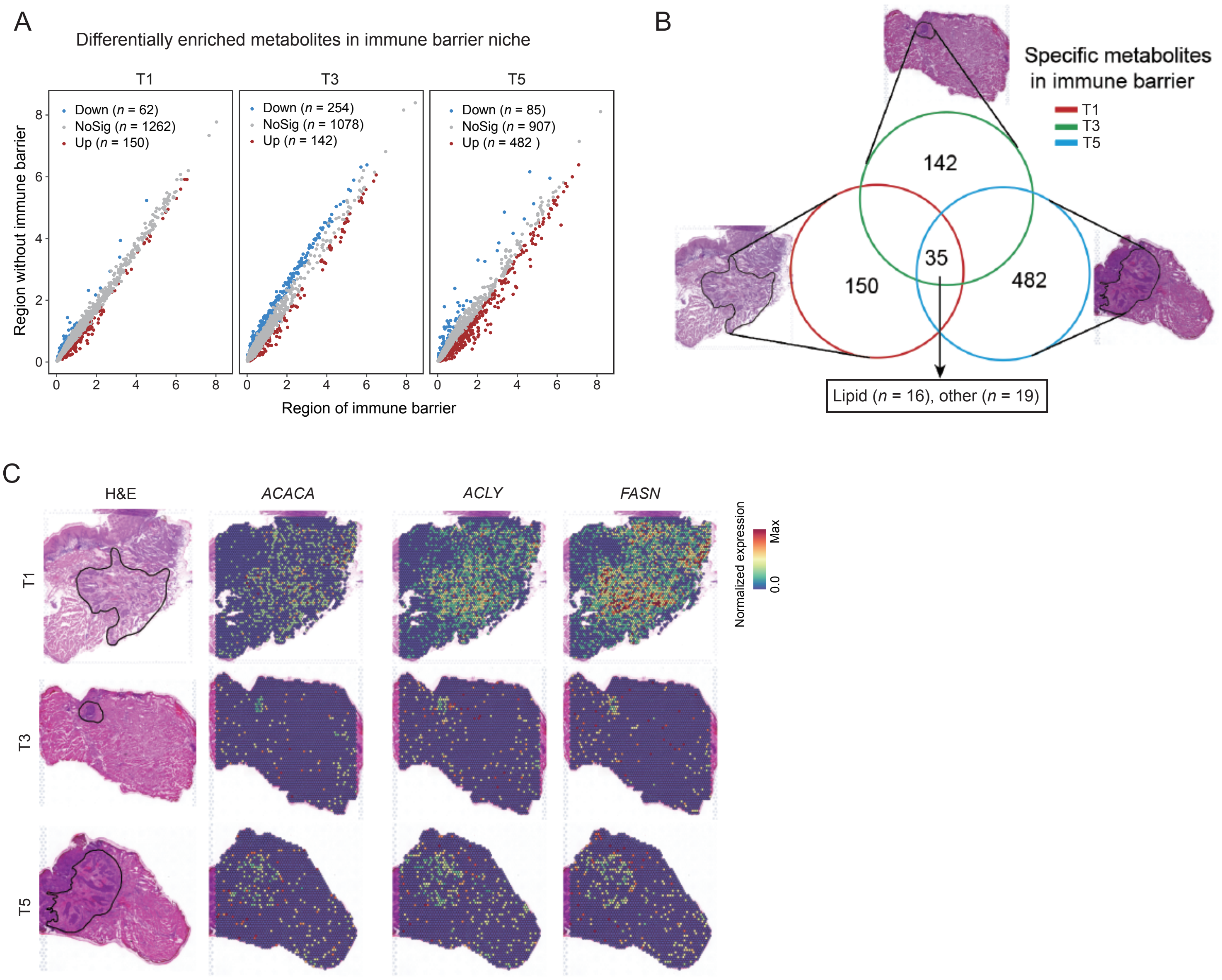
A
Differentially enriched metabolites in immune barrier niche
T1
T3
T5
Down (n = 62)
NoSig (n = 1262)
Up (n = 150)
Down (n = 254)
NoSig (n = 1078)
Up (n = 142)
Down (n = 85)
NoSig (n = 907)
Up (n = 482)
Region without immune barrier
Region of immune barrier
B
Specific metabolites in immune barrier
T1
T3
T5
142
150
35
482
Lipid (n = 16), other (n = 19)
C
H&E
ACACA
ACLY
FASN
T1
T3
T5
Normalized expression
Max
0.0
