# Supplementary material for: A Single-cell and Spatially Resolved Cell Atlas of Human Esophageal Squamous Cell Carcinoma
Source: Genomics Proteomics Bioinformatics. 2025 Nov 4;24(1):qzaf095. doi: 10.1093/gpbjnl/qzaf095 (PMC13433246; doi:10.1093/gpbjnl/qzaf095)
Supplement: Supplementary file 1 [file qzaf095_supplementary_data.zip › qzaf095_Supplementary_Data/FigS5.pdf]

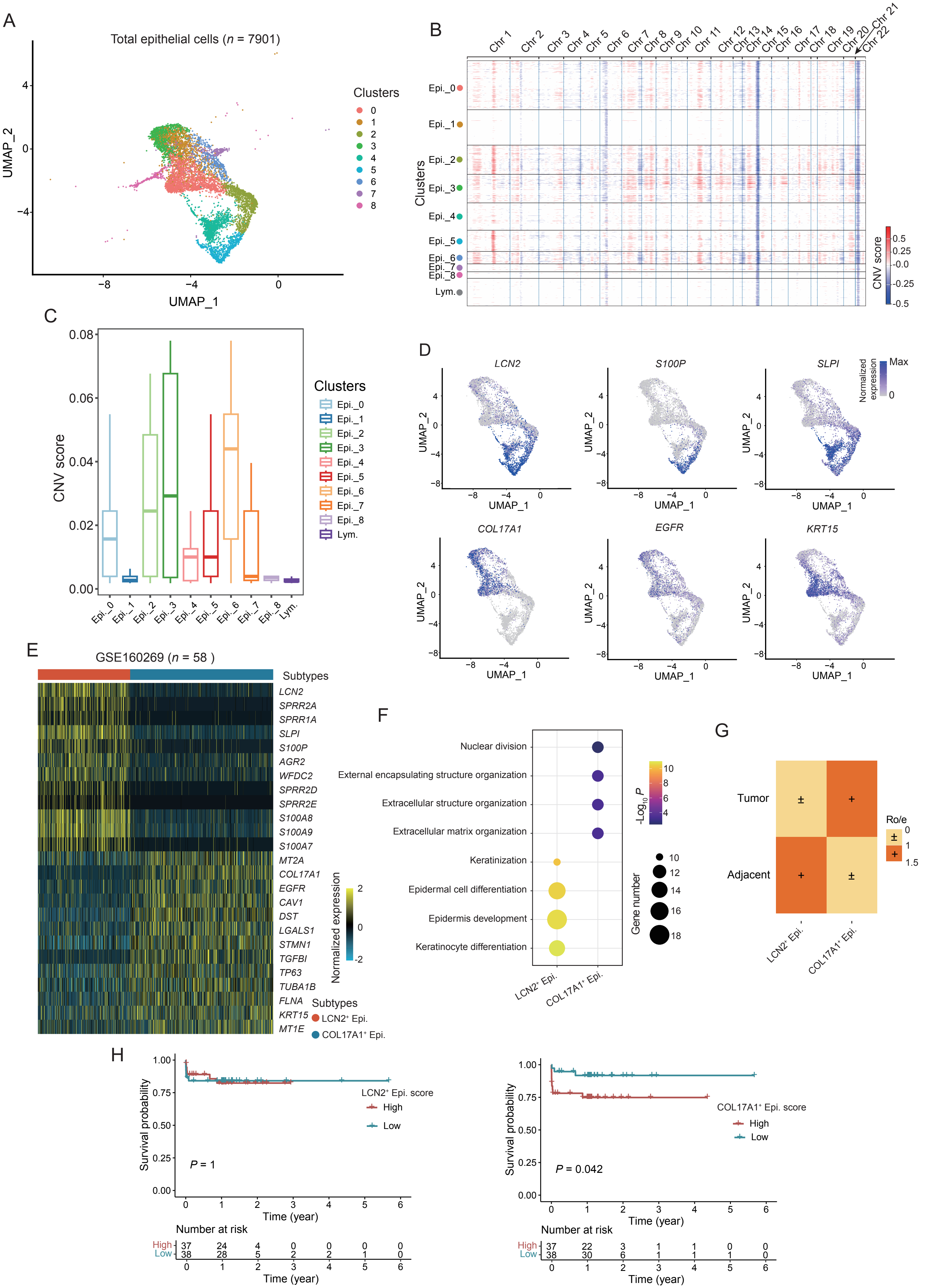

A
Total epithelial cells (n = 7901)
UMAP_1
UMAP_2
Clusters
B
Chr 1 Chr 2 Chr 3 Chr 4 Chr 5 Chr 6 Chr 7 Chr 8 Chr 9 Chr 10 Chr 11 Chr 12 Chr 13 Chr 14 Chr 15 Chr 16 Chr 17 Chr 18 Chr 19 Chr 20 Chr 21 Chr 22
Epi._0 Epi._1 Epi._2 Epi._3 Epi._4 Epi._5 Epi._6 Epi._7 Epi._8 Lym.
CNV score
C
CNV score
Clusters
D
LCN2 S100P SLPI COL17A1 EGFR KRT15
Normalized expression
Max
E
GSE160269 (n = 58)
Subtypes
LCN2 SPRR2A SPRR1A SLPI S100P AGR2 WFDC2 SPRR2D SPRR2E S100A8 S100A9 S100A7 MT2A COL17A1 EGFR CAV1 DST LGALS1 STMN1 TGFBI TP63 TUBA1B FLNA KRT15 MT1E
Normalized expression
LCN2⁺ Epi.
COL17A1⁺ Epi.
F
Nuclear division
External encapsulating structure organization
Extracellular structure organization
Extracellular matrix organization
Keratinization
Epidermal cell differentiation
Epidermis development
Keratinocyte differentiation
-Log10 P
Gene number
G
Tumor
Adjacent
Ro/e
H
LCN2⁺ Epi. score
High
Low
P = 1
Survival probability
Time (year)
Number at risk
COL17A1⁺ Epi. score
P = 0.042
