# Supplementary material for: A Single-cell and Spatially Resolved Cell Atlas of Human Esophageal Squamous Cell Carcinoma
Source: Genomics Proteomics Bioinformatics. 2025 Nov 4;24(1):qzaf095. doi: 10.1093/gpbjnl/qzaf095 (PMC13433246; doi:10.1093/gpbjnl/qzaf095)
Supplement: Supplementary file 1 [file qzaf095_supplementary_data.zip › qzaf095_Supplementary_Data/figS7.pdf]

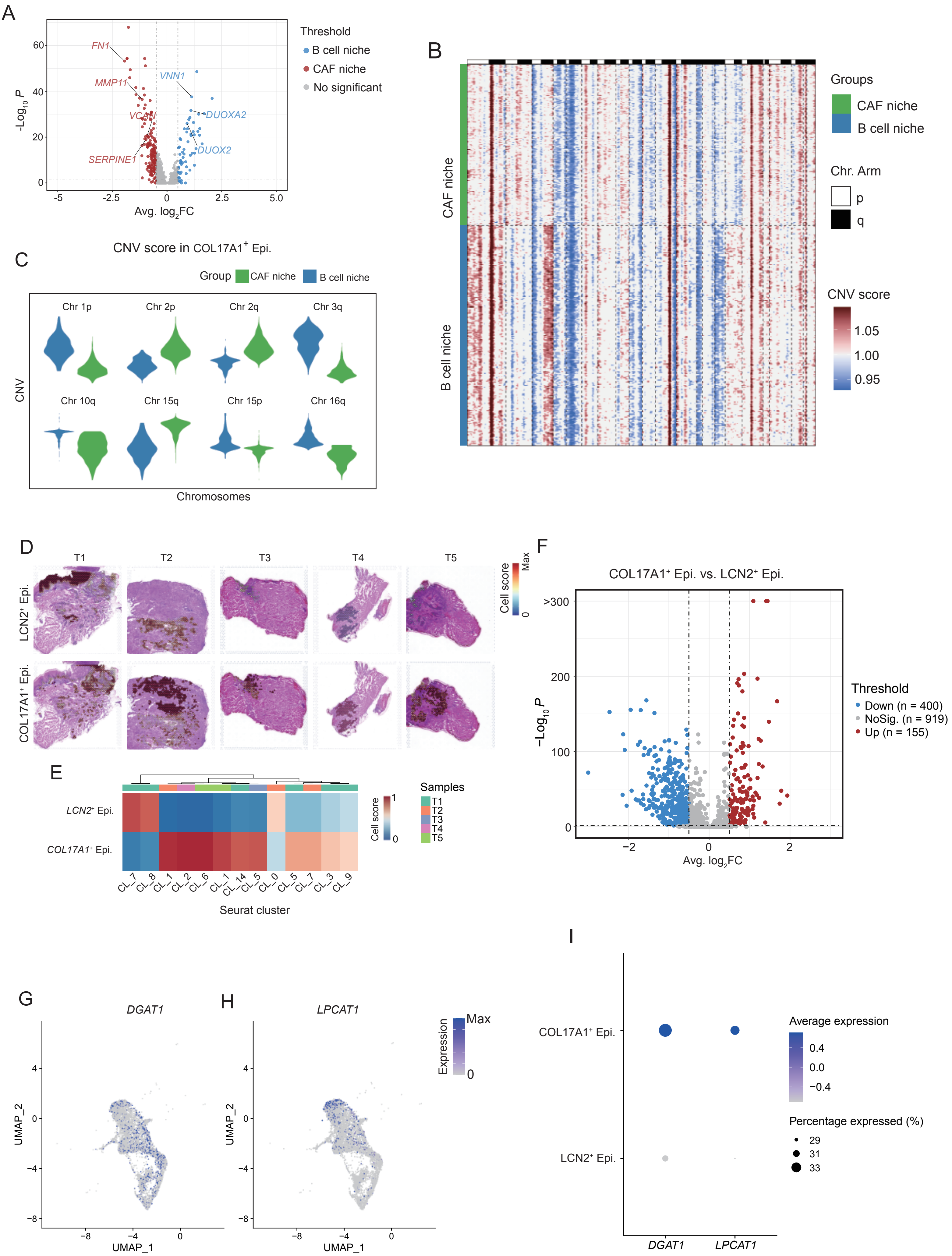

A
FN1
MMP11
VCAN
SERPINE1
VNN1
DUOXA2
DUOX2
-Log10 P
Avg. log2FC
Threshold
B cell niche
CAF niche
No significant
B
CAF niche
B cell niche
Groups
CAF niche
B cell niche
Chr. Arm
p
q
CNV score
1.05
1.00
0.95
C
CNV score in COL17A1+ Epi.
Group
CAF niche
B cell niche
Chr 1p
Chr 2p
Chr 2q
Chr 3q
Chr 10q
Chr 15q
Chr 15p
Chr 16q
CNV
Chromosomes
D
T1
T2
T3
T4
T5
LCN2+ Epi.
COL17A1+ Epi.
Cell score
Max
0
E
LCN2+ Epi.
COL17A1+ Epi.
Cell score
Samples
T1
T2
T3
T4
T5
CL_7
CL_8
CL_1
CL_2
CL_6
CL_1
CL_14
CL_5
CL_0
CL_5
CL_7
CL_3
CL_9
Seurat cluster
F
COL17A1+ Epi. vs. LCN2+ Epi.
>300
200
100
0
−Log10 P
Avg. log2FC
Threshold
Down (n = 400)
NoSig. (n = 919)
Up (n = 155)
G
DGAT1
UMAP_2
UMAP_1
H
LPCAT1
Expression
Max
0
I
COL17A1+ Epi.
LCN2+ Epi.
DGAT1
LPCAT1
Average expression
0.4
0.0
−0.4
Percentage expressed (%)
29
31
33
