# Supplementary material for: A Single-cell and Spatially Resolved Cell Atlas of Human Esophageal Squamous Cell Carcinoma
Source: Genomics Proteomics Bioinformatics. 2025 Nov 4;24(1):qzaf095. doi: 10.1093/gpbjnl/qzaf095 (PMC13433246; doi:10.1093/gpbjnl/qzaf095)
Supplement: Supplementary file 1 [file qzaf095_supplementary_data.zip › qzaf095_Supplementary_Data/FigS6.pdf]

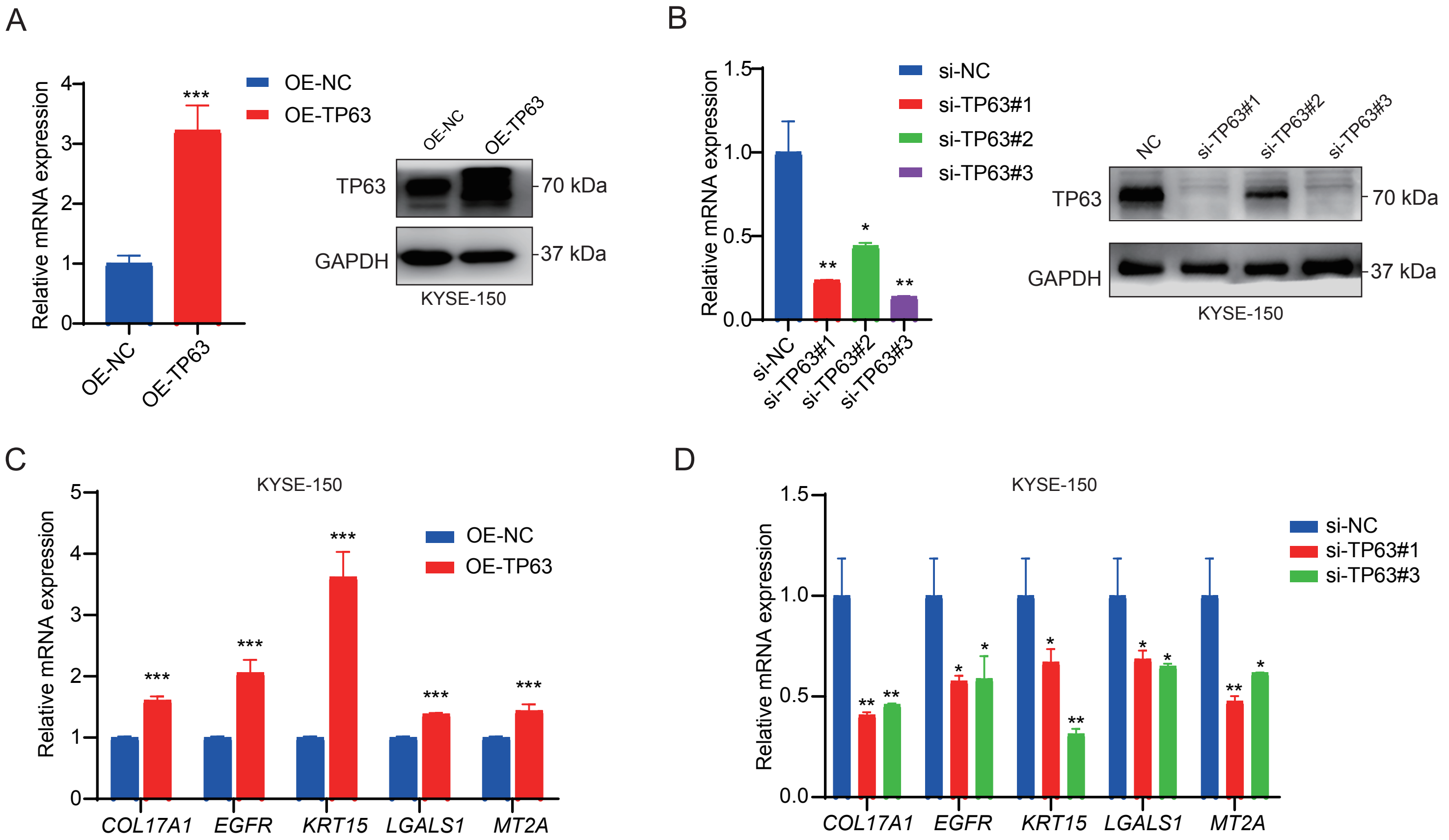
A
Relative mRNA expression
OE-NC
OE-TP63
***
TP63
70 kDa
GAPDH
37 kDa
KYSE-150
B
si-NC
si-TP63#1
si-TP63#2
si-TP63#3
NC
TP63
70 kDa
GAPDH
37 kDa
KYSE-150
C
KYSE-150
COL17A1
EGFR
KRT15
LGALS1
MT2A
D
KYSE-150
COL17A1
EGFR
KRT15
LGALS1
MT2A
