# Supplementary material for: A Single-cell and Spatially Resolved Cell Atlas of Human Esophageal Squamous Cell Carcinoma
Source: Genomics Proteomics Bioinformatics. 2025 Nov 4;24(1):qzaf095. doi: 10.1093/gpbjnl/qzaf095 (PMC13433246; doi:10.1093/gpbjnl/qzaf095)
Supplement: Supplementary file 1 [file qzaf095_supplementary_data.zip › qzaf095_Supplementary_Data/figS2.pdf]

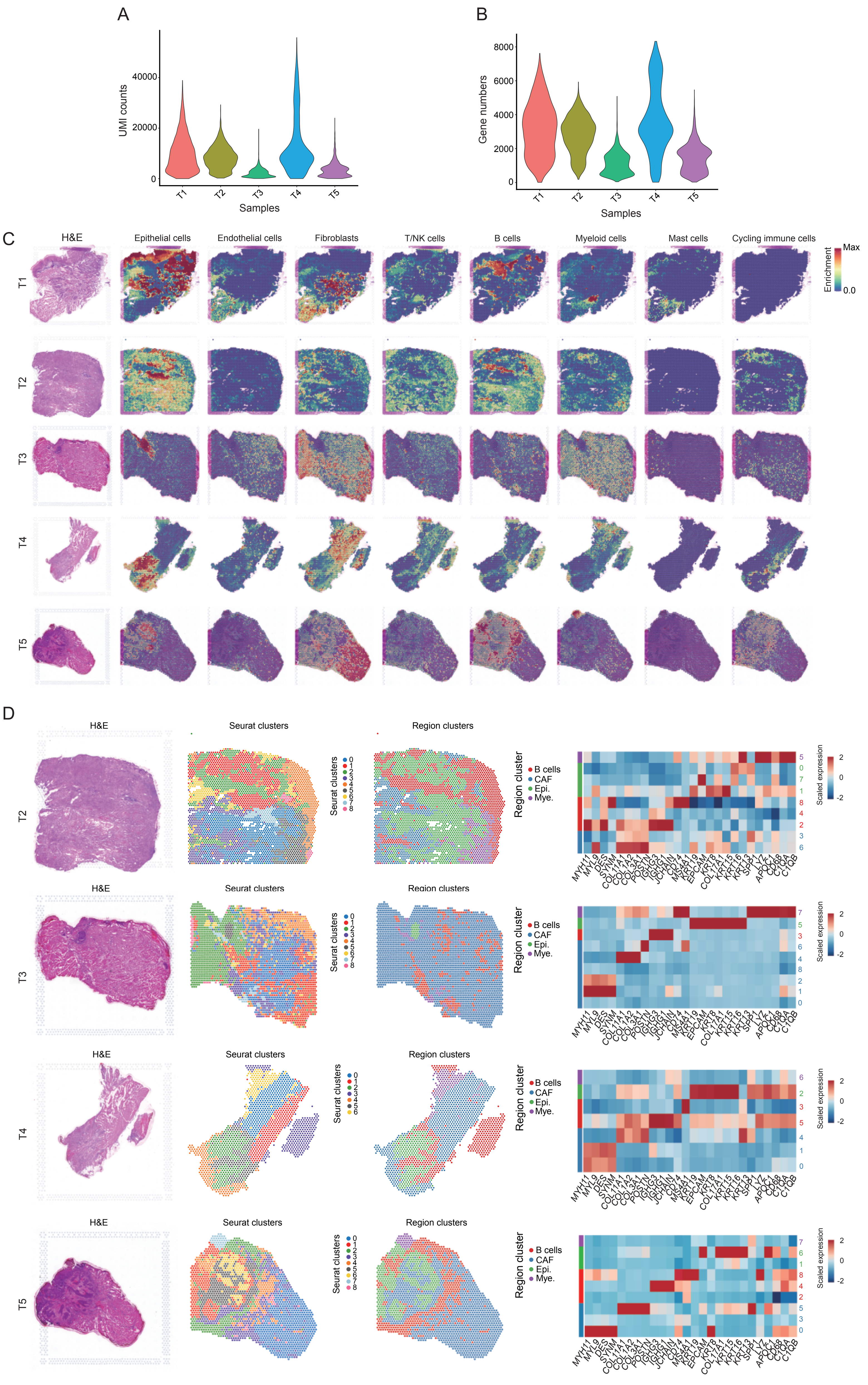
A
UMI counts
40000
20000
0
T1
T2
T3
T4
T5
Samples
B
Gene numbers
8000
6000
4000
2000
0
T1
T2
T3
T4
T5
Samples
C
H&E
Epithelial cells
Endothelial cells
Fibroblasts
T/NK cells
B cells
Myeloid cells
Mast cells
Cycling immune cells
Enrichment
Max
0.0
T1
T2
T3
T4
T5
D
H&E
Seurat clusters
Region clusters
Region cluster
B cells
CAF
Epi.
Mye.
Scaled expression
2
0
-2
T2
T3
T4
T5
MYH11
MYL9
DES
SYNM
COL11A1
COL1A2
COL3A1
POSTN
IGHG3
IGHG1
JCHAIN
CD74
MS4A1
KRT19
EPCAM
KRT8
COL17A1
KRT15
KRT16
KRT13
SPP1
LYZ
APOC1
CD68
C1QA
C1QB
