# Supplementary material for: A Single-cell and Spatially Resolved Cell Atlas of Human Esophageal Squamous Cell Carcinoma
Source: Genomics Proteomics Bioinformatics. 2025 Nov 4;24(1):qzaf095. doi: 10.1093/gpbjnl/qzaf095 (PMC13433246; doi:10.1093/gpbjnl/qzaf095)
Supplement: Supplementary file 1 [file qzaf095_supplementary_data.zip › qzaf095_Supplementary_Data/FigS3.pdf]

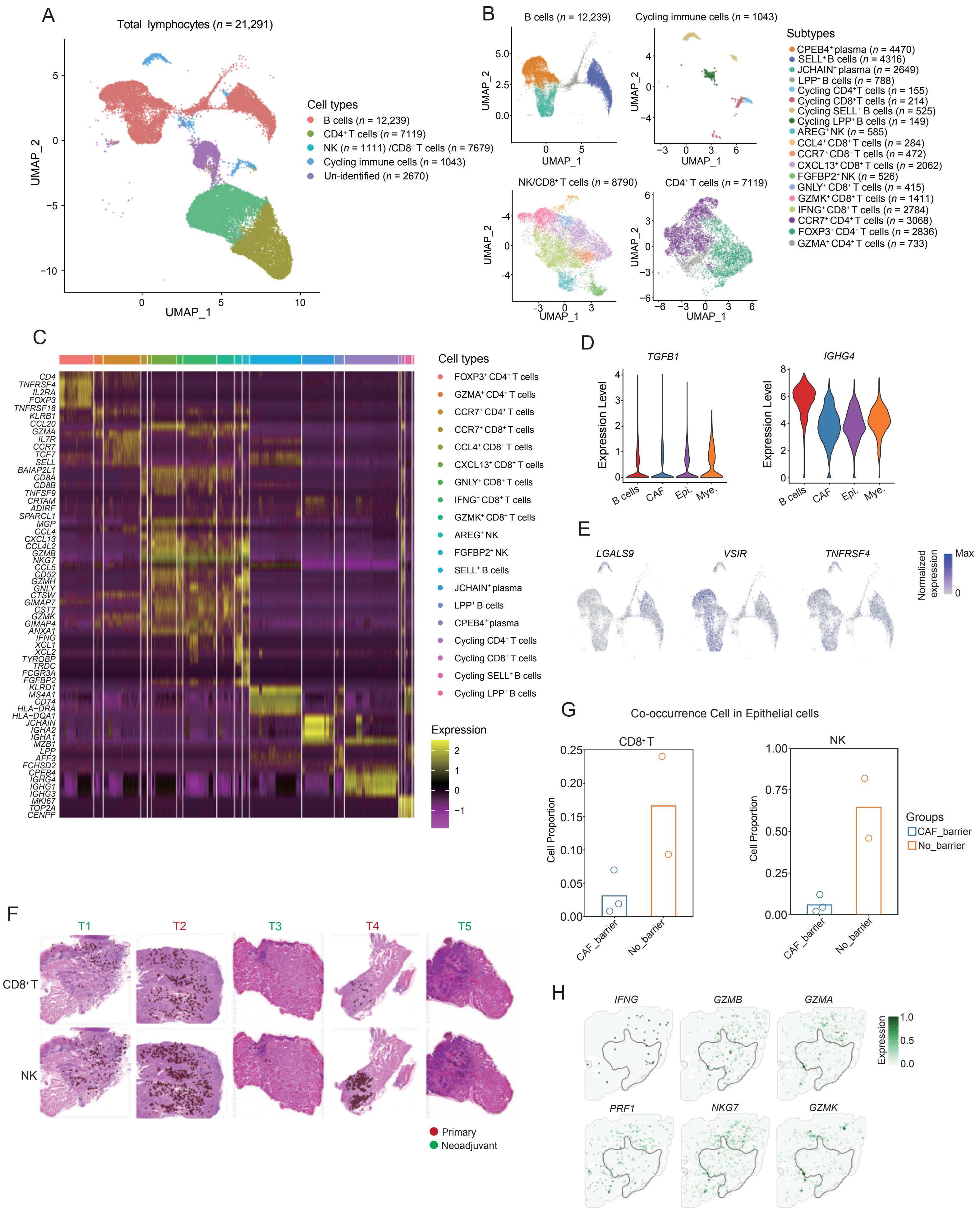

A
Total lymphocytes (n = 21,291)
UMAP_1
UMAP_2
Cell types
B cells (n = 12,239)
CD4⁺ T cells (n = 7119)
NK (n = 1111) /CD8⁺ T cells (n = 7679)
Cycling immune cells (n = 1043)
Un-identified (n = 2670)
B
B cells (n = 12,239)
Cycling immune cells (n = 1043)
NK/CD8⁺ T cells (n = 8790)
CD4⁺ T cells (n = 7119)
Subtypes
CPEB4⁺ plasma (n = 4470)
SELL⁺ B cells (n = 4316)
JCHAIN⁺ plasma (n = 2649)
LPP⁺ B cells (n = 788)
Cycling CD4⁺T cells (n = 155)
Cycling CD8⁺T cells (n = 214)
Cycling SELL⁺ B cells (n = 525)
Cycling LPP⁺ B cells (n = 149)
AREG⁺ NK (n = 585)
CCL4⁺ CD8⁺ T cells (n = 284)
CCR7⁺ CD8⁺ T cells (n = 472)
CXCL13⁺ CD8⁺ T cells (n = 2062)
FGFBP2⁺ NK (n = 526)
GNLY⁺ CD8⁺ T cells (n = 415)
GZMK⁺ CD8⁺ T cells (n = 1411)
IFNG⁺ CD8⁺ T cells (n = 2784)
CCR7⁺ CD4⁺ T cells (n = 3068)
FOXP3⁺ CD4⁺ T cells (n = 2836)
GZMA⁺ CD4⁺ T cells (n = 733)
C
Cell types
FOXP3⁺ CD4⁺ T cells
GZMA⁺ CD4⁺ T cells
CCR7⁺ CD4⁺ T cells
CCR7⁺ CD8⁺ T cells
CCL4⁺ CD8⁺ T cells
CXCL13⁺ CD8⁺ T cells
GNLY⁺ CD8⁺ T cells
IFNG⁺ CD8⁺ T cells
GZMK⁺ CD8⁺ T cells
AREG⁺ NK
FGFBP2⁺ NK
SELL⁺ B cells
JCHAIN⁺ plasma
LPP⁺ B cells
CPEB4⁺ plasma
Cycling CD4⁺ T cells
Cycling CD8⁺ T cells
Cycling SELL⁺ B cells
Cycling LPP⁺ B cells
Expression
CD4 TNFRSF4 IL2RA FOXP3 TNFRSF18 KLRB1 CCL20 GZMA IL7R CCR7 TCF7 SELL BAIAP2L1 CD8A CD8B TNFSF9 CRTAM ADIRF SPARCL1 MGP CCL4 CXCL13 CCL4L2 GZMB NKG7 CCL5 CD52 GZMH GNLY CTSW GIMAP7 CST7 GZMK GIMAP4 ANXA1 IFNG XCL1 XCL2 TYROBP TRDC FCGR3A FGFBP2 KLRD1 MS4A1 CD74 HLA−DRA HLA−DQA1 JCHAIN IGHA2 IGHA1 MZB1 LPP AFF3 FCHSD2 CPEB4 IGHG4 IGHG1 IGHG3 MKI67 TOP2A CENPF
D
TGFB1
IGHG4
Expression Level
B cells CAF Epi. Mye.
E
LGALS9
VSIR
TNFRSF4
Normalized expression
Max
0
F
T1 T2 T3 T4 T5
CD8⁺ T
NK
Primary
Neoadjuvant
G
Co-occurrence Cell in Epithelial cells
CD8⁺ T
NK
Cell Proportion
Groups
CAF_barrier
No_barrier
H
IFNG GZMB GZMA
PRF1 NKG7 GZMK
Expression
