# Supplementary material for: A Single-cell and Spatially Resolved Cell Atlas of Human Esophageal Squamous Cell Carcinoma
Source: Genomics Proteomics Bioinformatics. 2025 Nov 4;24(1):qzaf095. doi: 10.1093/gpbjnl/qzaf095 (PMC13433246; doi:10.1093/gpbjnl/qzaf095)
Supplement: Supplementary file 1 [file qzaf095_supplementary_data.zip › qzaf095_Supplementary_Data/FigS1.pdf]

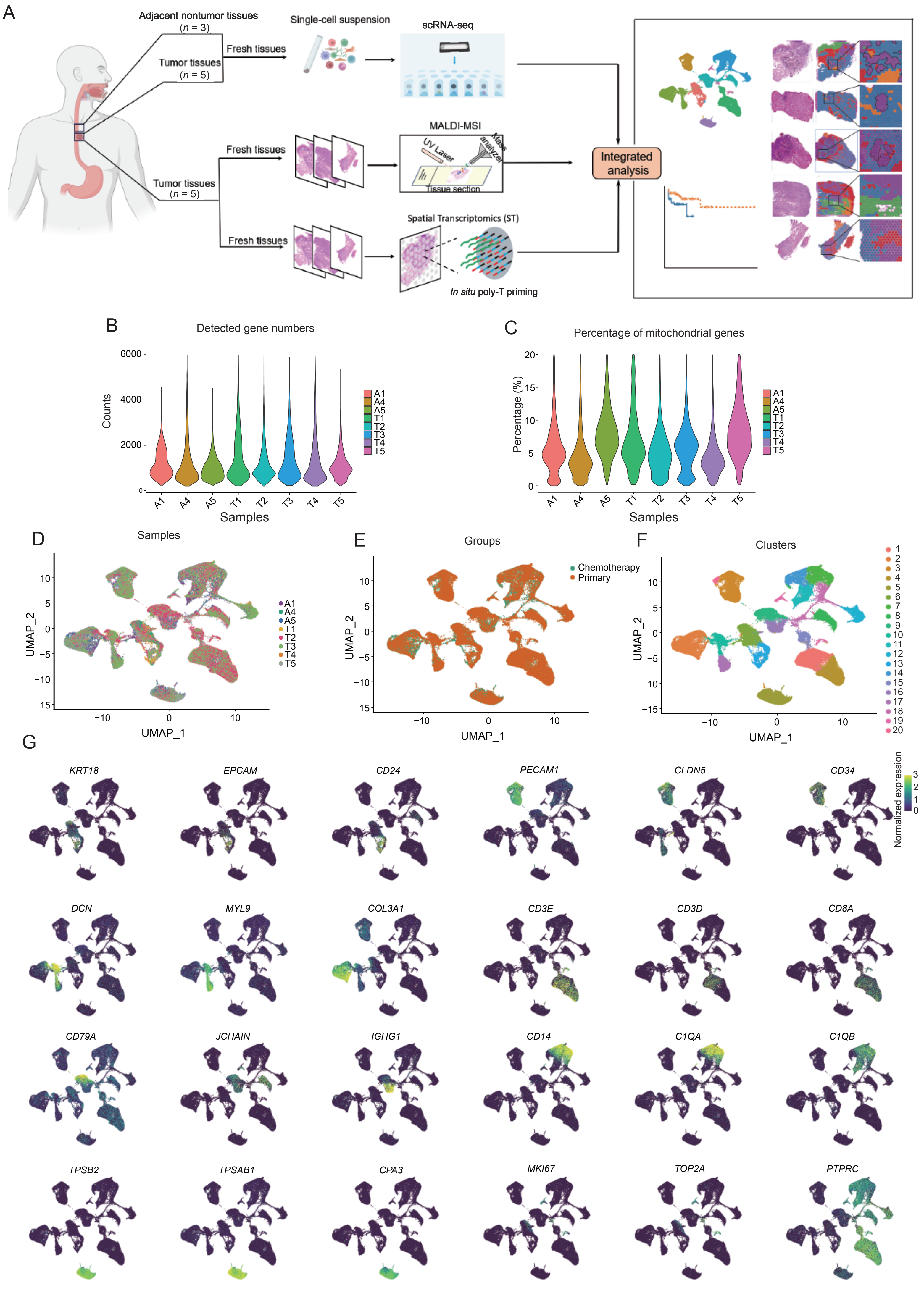
A
Adjacent nontumor tissues
(n = 3)
Fresh tissues
Tumor tissues
(n = 5)
Single-cell suspension
scRNA-seq
MALDI-MSI
UV Laser
Mass analyzer
Tissue section
Tumor tissues
(n = 5)
Fresh tissues
Fresh tissues
Integrated analysis
Spatial Transcriptomics (ST)
In situ poly-T priming
B
Detected gene numbers
Counts
Samples
A1 A4 A5 T1 T2 T3 T4 T5
C
Percentage of mitochondrial genes
Percentage (%)
Samples
A1 A4 A5 T1 T2 T3 T4 T5
D
Samples
UMAP_1
UMAP_2
E
Groups
Chemotherapy
Primary
F
Clusters
1 2 3 4 5 6 7 8 9 10 11 12 13 14 15 16 17 18 19 20
G
KRT18
EPCAM
CD24
PECAM1
CLDN5
CD34
Normalized expression
DCN
MYL9
COL3A1
CD3E
CD3D
CD8A
CD79A
JCHAIN
IGHG1
CD14
C1QA
C1QB
TPSB2
TPSAB1
CPA3
MKI67
TOP2A
PTPRC
