# Supplementary material for: A Single-cell and Spatially Resolved Cell Atlas of Human Esophageal Squamous Cell Carcinoma
Source: Genomics Proteomics Bioinformatics. 2025 Nov 4;24(1):qzaf095. doi: 10.1093/gpbjnl/qzaf095 (PMC13433246; doi:10.1093/gpbjnl/qzaf095)
Supplement: Supplementary file 1 [file qzaf095_supplementary_data.zip › qzaf095_Supplementary_Data/Table_S1.docx]

| **Table S1 Basic clinical information of patients included in this study** | | | | | | | |
| --- | --- | --- | --- | --- | --- | --- | --- |
| **ID** | **Age (years)** | **Sex** | **Diagnosis** | **TNM** | **Matched-adjacent cancerous tissues** | **Neoadjuvent chemotherapy** | **Pathological grading** |
| 1 | 67 | Male | ESCC | pT3N1MX | Yes | Karelizumab+cisplatin+paclitaxel | II |
| 2 | 84 | Male | ESCC | Not reported | No | No | II-III |
| 3 | 65 | Female | ESCC | pT1aN0MX | No | Karelizumab+cisplatin+paclitaxel | II |
| 4 | 68 | Female | ESCC | pT3N1MX | Yes | No | II |
| 5 | 66 | Male | ESCC | T3-T4 | No | Karelizumab+cisplatin+paclitaxel | Not reported |
