# Supplementary material for: A Single-cell and Spatially Resolved Cell Atlas of Human Esophageal Squamous Cell Carcinoma
Source: Genomics Proteomics Bioinformatics. 2025 Nov 4;24(1):qzaf095. doi: 10.1093/gpbjnl/qzaf095 (PMC13433246; doi:10.1093/gpbjnl/qzaf095)
Supplement: Supplementary file 1 [file qzaf095_supplementary_data.zip › qzaf095_Supplementary_Data/Supplementary material captions 092725.docx]

**Supplementary material**

**Figure S1 Single cell clustering and cell proportion analysis**

**A.** Workflow of multiomics integration analyses for scRNA-seq, ST and SM. **B.** Violin plots showing average counts of detected genes in each ESCC sample. **C.** Violin plots showing the percent of mitochondrial genes across all samples after quality control. **D.** UMAP plots of total cells labeled by samples. **E.** UMAP plots of total cells labeled by groups. **F.** UMAP plots of total cells labeled by seurat clusters. **G.** Feature plots of marker genes for eight major cell types.

**Figure S2 Integration of single-cell and spatial transcriptomic data**

**A.** and **B.** Violin plots showing the average UMI counts (A) and detected genes (B) across five sections in ST data. **C.** Spatial feature plots of enrichment scores for eight ESCC cell populations across five tumor samples. **D.** Left: H&E staining of tissue sections, unbiased clustering of ST spots and four spatial niche clusters. Right: heatmap showing the expression of marker genes for four spatial niches. UMI, unique molecular identifier.

**Figure S3 The features of tumor microenvironment in ESCC**

**A.** UMAP plots of major lymphocyte populations. **B.** UMAP plots of subpopulations for B cells, cycling immune cells, CD8^+^ T cells, CD4^+^ T cells. **C.** Heatmap showing the mRNA expression of maker genes for all lymphocyte subpopulations. **D.** Violin plots showing the mRNA expression of *TGFB1* and *IGHG4* across four spatial niches in ST data. **E.** Feature plots of *LGALS9*, *VSIR*, *TNFRSF4* for B cells in scRNA-seq data. **F.** H&E staining with co-occurrence spots of CD8^+^ T/NK and epithelial cells. **G.** Box plots showing the proportion of co-occurrence spots of CD8^+^ T/NK and epithelial cells in total tumor spots between CAF barrier and no barrier groups. **H.** Spatial feature plots of selected immune effector genes including *IFNG*, *GZMB*, *GZMA*, *PRF1*, *NKG7*, *GZMK* in tumor sample 1.

**Figure S4 Identification of metabolic features in the immune barrier region**

**A.** Volcano plots of differential metabolites (|log_2_-fold change| > 1 and *P* < 0.05) between the CAF barrier and non-barrier regions in tumor samples 1, 3, and 5. **B.** Venn diagrams illustrating the intersection of highly enriched metabolites in the immune barrier region across tumor samples 1, 3, and 5. **C.** Spatial feature plots displaying the spatial distribution of lipid synthesis-related genes *ACACA*, *ACLY*, and *FASN* in tumor samples 1, 3, and 5.

**Figure S5 Epithelial cell clustering and functional analysis**

**A.** UMAP plots of total epithelial cells labeled by seurat clusters. **B.** Heatmap showing the genomic CNV scores of nine epithelial cell clusters. **C.** Boxplots showing the mean CNV scores across epithelial subpopulations. **D.** Feature plots of represented marker genes *LCN2*, *S100P*, *SLPI*, *KRT15*, *COL17A1*, and *EGFR*. **E.** Heatmap showing the expression of 26 signature genes in epithelial cells from GSE160269 dataset (*n* = 58). **F.** Dot plots showing enriched GO terms for the top 100 upregulated genes in each epithelial subpopulation. **G.** Heatmap showing cell preference distribution of LCN2^+^ or COL17A1^+^ epithelial cells in paired samples (*n* = 3), with Ro/e < 1 indicating nontumor-preferential distribution and Ro/e > 1 indicating tumor-preferential distribution. **H.** Kaplan–Meier survival curve for LCN2^+^ or COL17A1^+^ epithelial cell signature score in a TCGA-ESCC cohort (*n* = 75). Statistical significance is set at *P* < 0.05 (Wilcoxon test).

**Figure S6 Validation of *TP63* overexpression and knockdown efficiency in KYSE-150**

**A.** Quantitative real-time polymerase chain reaction (qPCR) and WB analysis of *TP63* expression levels in KYSE-150 cells transfected with *TP63*-overexpressing lentivirus after 10 days. **B.** qPCR and WB analysis of *TP63* expression levels in KYSE-150 cells transfected with si-TP63 after 48 h. **C.** and **D.** qPCR analysis of genes *COL17A1*, *MT2A*, *EGFR*, *KRT15*, and *LGALS1* after *TP63* overexpression (C) or knockdown (D) in KYSE-150 cells. ns, *P* is not significant; *, *P* < 0.05; **, *P* < 0.01; ***, *P* < 0.001. Statistical significance was calculated by Student’s *t*-test, mean ± SD.

**Figure S7 Spatial characteristics of epithelial subpopulations**

**A.** Volcano plots showing differentially expressed genes for COL17A1^+^ epithelial cells between B cell and CAF niches in tumor sample 1. *P* < 0.05 and log_2_-fold change > 0.5 were considered as a statistically significant difference. **B.** Heatmap showing the genomic CNV scores of COL17A1^+^ epithelial cells in B cell or CAF niches. **C.** Violin plots showing the CNV score changes of representative chromosomes between B cell and CAF niches. **D.** Spatial feature plots showing the abundance of epithelial subpopulations in epithelial region across five tumor samples. **E.** Heatmap displaying the average abundance of epithelial subpopulations in seurat clusters across five tumor samples. **F.** Volcano plots showing differentially enriched metabolites between LCN2^+^ and COL17A1^+^ epithelial cells. *P* < 0.05 and log_2_-fold change > 0.5 are considered as a statistically significant difference. **G.** and **H.** Feature plots of *DGAT1* (G) and *LPACAT1* (H) for epithelial subpopulations in scRNA-seq data. **I.** Dot plots showing the mRNA expression of *DGAT1* and *LPACAT1* in epithelial subpopulations from ST data.

**Figure S8 Validation of *TWIST1* overexpression and knockdown efficiency in BJ cells**

**A.** and **B.** UMAP plots of total fibroblasts labeled by groups (A) and samples (B). **C.** Bar plots showing fibroblast subcluster proportions across eight samples. **D.** GO terms of top 100 specifically expressed genes for each fibroblast subpopulation. **E.** qPCR and WB analysis of *TWIST1* expression levels in BJ cells transfected with *TWIST1* overexpression lentivirus after 10 days. **F.** qPCR and WB analysis of *TWIST1* expression levels in BJ cells transfected with si-TWIST1 after 48 h. **G.** and **H.** qPCR analysis of genes *POSTN*, *COL1A1*, *COL3A1*, *INHBA*, *COL5A2* after *TWIST1* overexpression (G) or knockdown (H) in BJ cells. *, *P* < 0.05; **, *P* < 0.01; ***, *P* < 0.001. Statistical significance was calculated by Student’s *t*-test, mean ± SD.

**Figure S9 Representative cell–cell communication between tumor cells and fibroblasts**

**A.** H&E staining overlays mRNA expression for selected L-R pairs (COL17A1^+^ epithelial cells to POSTN^+^/CFD^+^ fibroblasts) across tumor samples 1,3, and 5. **B.** H&E staining overlays mRNA expression for selected L-R pairs (POSTN^+^ fibroblasts to COL17A1^+^ epithelial cells) across tumor samples 1,3, and 5. **C.** Feature plots of scRNA-seq expression for *JAG1* and *NOTCH2*. **E.** The network of top active ligands from POSTN^+^ fibroblasts, matched receptors and matched target genes from COL17A1^+^ epithelial cells. Red represents ligands; orange represents receptors; green represents target genes. **D.** and **F.** Represented GO terms of target genes expressed by POSTN^+^ fibroblasts (D) or COL17A1^+^ epithelial cells (F).

**Figure S10 Analysis of *INHBA* expression levels and prognosis in public database**

**A.** Dot plots showing the mRNA expression of *INHBA* across eight major cell types in scRNA-seq data. **B.** Box plots showing *INHBA* expression between ESCC (*n* = 95) and normal tissues (*n* = 13) in TCGA. **C.** Scatter plots showing the correlation between *INHBA* and *TP63* mRNA expression levels in TCGA-ESCC dataset (*n* = 95).

**Table S1 Baseline information of clinical pathological parameters in five samples with ESCC**
