# Supplementary material for: A Single-cell and Spatially Resolved Cell Atlas of Human Esophageal Squamous Cell Carcinoma
Source: Genomics Proteomics Bioinformatics. 2025 Nov 4;24(1):qzaf095. doi: 10.1093/gpbjnl/qzaf095 (PMC13433246; doi:10.1093/gpbjnl/qzaf095)
Supplement: Supplementary file 1 [file qzaf095_supplementary_data.zip › qzaf095_Supplementary_Data/FigS8.pdf]

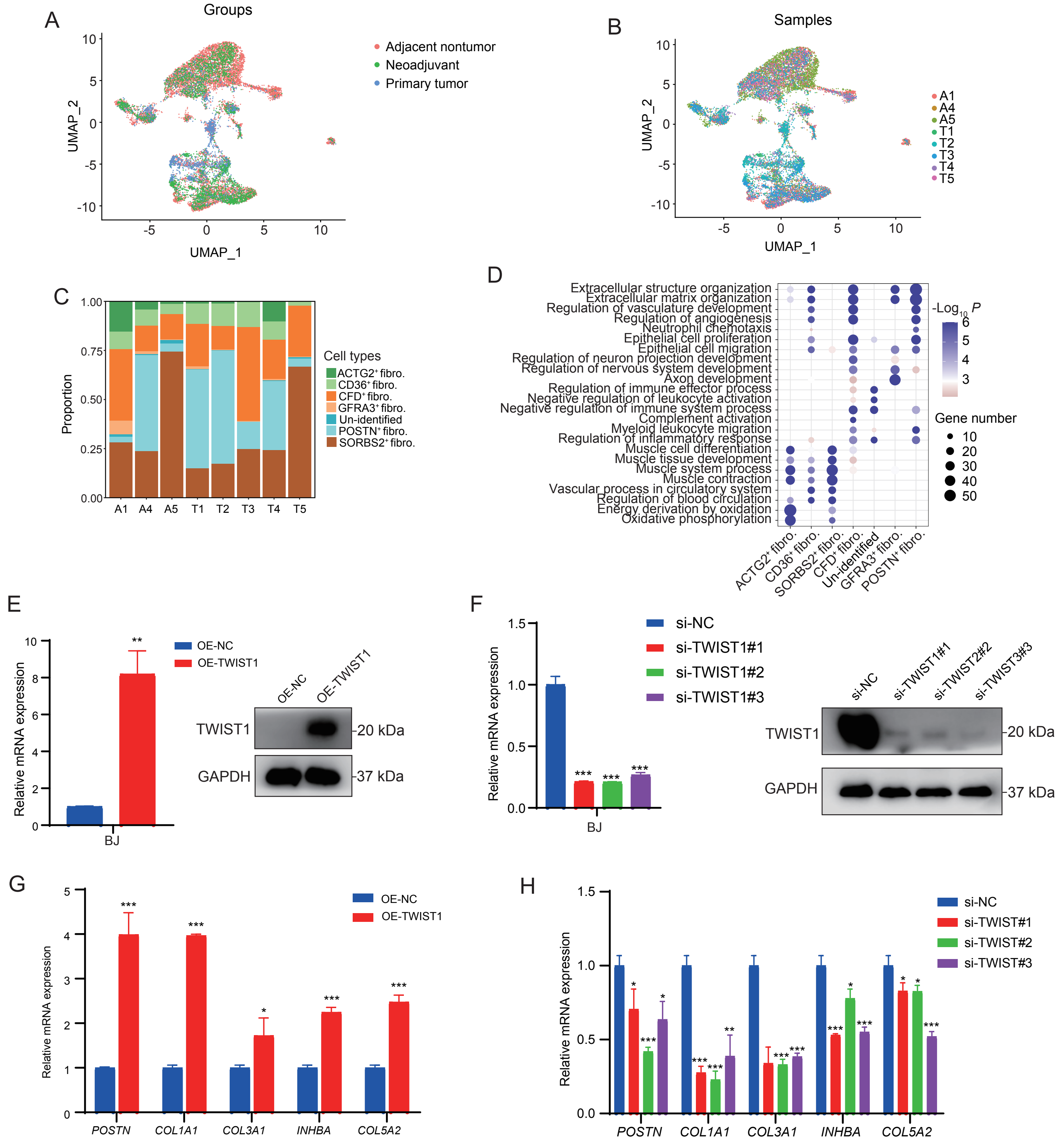
A
Groups
Adjacent nontumor
Neoadjuvant
Primary tumor
UMAP_1
UMAP_2
B
Samples
A1
A4
A5
T1
T2
T3
T4
T5
C
Proportion
Cell types
ACTG2+ fibro.
CD36+ fibro.
CFD+ fibro.
GFRA3+ fibro.
Un-identified
POSTN+ fibro.
SORBS2+ fibro.
D
Extracellular structure organization
Extracellular matrix organization
Regulation of vasculature development
Regulation of angiogenesis
Neutrophil chemotaxis
Epithelial cell proliferation
Epithelial cell migration
Regulation of neuron projection development
Regulation of nervous system development
Axon development
Regulation of immune effector process
Negative regulation of leukocyte activation
Negative regulation of immune system process
Complement activation
Myeloid leukocyte migration
Regulation of inflammatory response
Muscle cell differentiation
Muscle tissue development
Muscle system process
Muscle contraction
Vascular process in circulatory system
Regulation of blood circulation
Energy derivation by oxidation
Oxidative phosphorylation
-Log10 P
Gene number
E
Relative mRNA expression
OE-NC
OE-TWIST1
BJ
TWIST1
GAPDH
20 kDa
37 kDa
F
si-NC
si-TWIST1#1
si-TWIST1#2
si-TWIST1#3
si-TWIST2#2
si-TWIST3#3
G
POSTN
COL1A1
COL3A1
INHBA
COL5A2
H
si-TWIST#1
si-TWIST#2
si-TWIST#3
