# Supplementary material for: A Single-cell and Spatially Resolved Cell Atlas of Human Esophageal Squamous Cell Carcinoma
Source: Genomics Proteomics Bioinformatics. 2025 Nov 4;24(1):qzaf095. doi: 10.1093/gpbjnl/qzaf095 (PMC13433246; doi:10.1093/gpbjnl/qzaf095)
Supplement: Supplementary file 1 [file qzaf095_supplementary_data.zip › qzaf095_Supplementary_Data/FigS10.pdf]

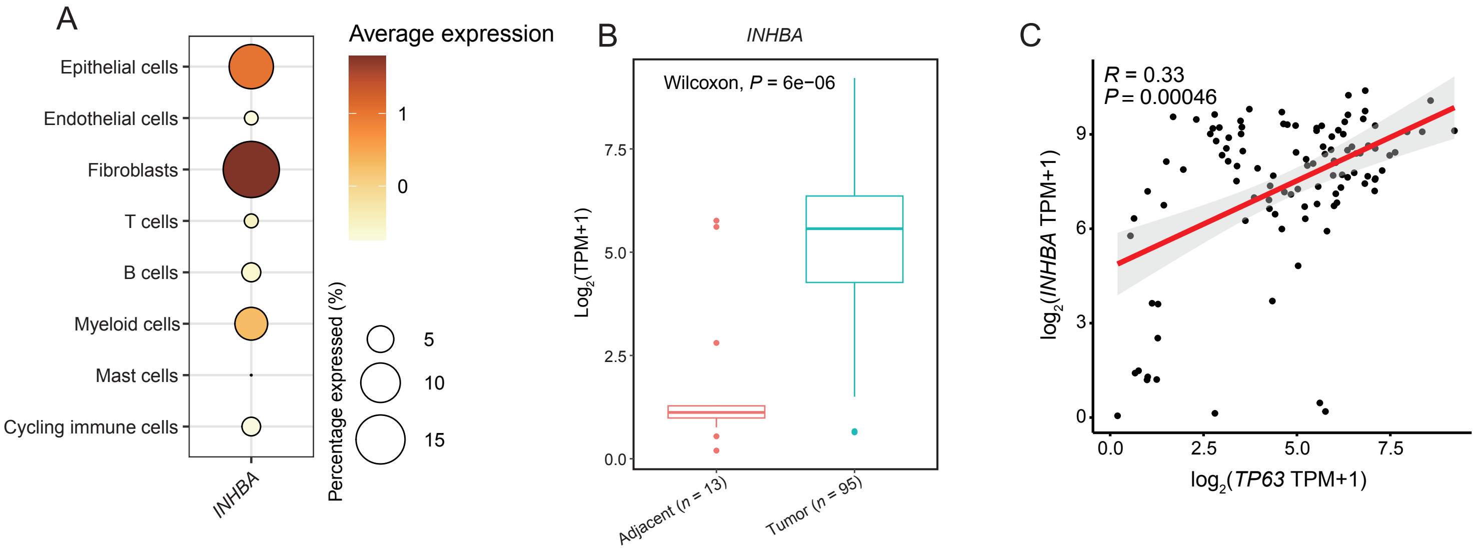

A
Average expression
1
0
Epithelial cells
Endothelial cells
Fibroblasts
T cells
B cells
Myeloid cells
Mast cells
Cycling immune cells
INHBA
Percentage expressed (%)
5
10
15
B
INHBA
Wilcoxon, P = 6e−06
Log2(TPM+1)
7.5
5.0
2.5
0.0
Adjacent (n = 13)
Tumor (n = 95)
C
R = 0.33
P = 0.00046
9
6
3
0
log2(INHBA TPM+1)
0.0
2.5
5.0
7.5
log2(TP63 TPM+1)
