# Supplementary material for: A Single-cell and Spatially Resolved Cell Atlas of Human Esophageal Squamous Cell Carcinoma
Source: Genomics Proteomics Bioinformatics. 2025 Nov 4;24(1):qzaf095. doi: 10.1093/gpbjnl/qzaf095 (PMC13433246; doi:10.1093/gpbjnl/qzaf095)
Supplement: Supplementary file 1 [file qzaf095_supplementary_data.zip › qzaf095_Supplementary_Data/FigS9.pdf]

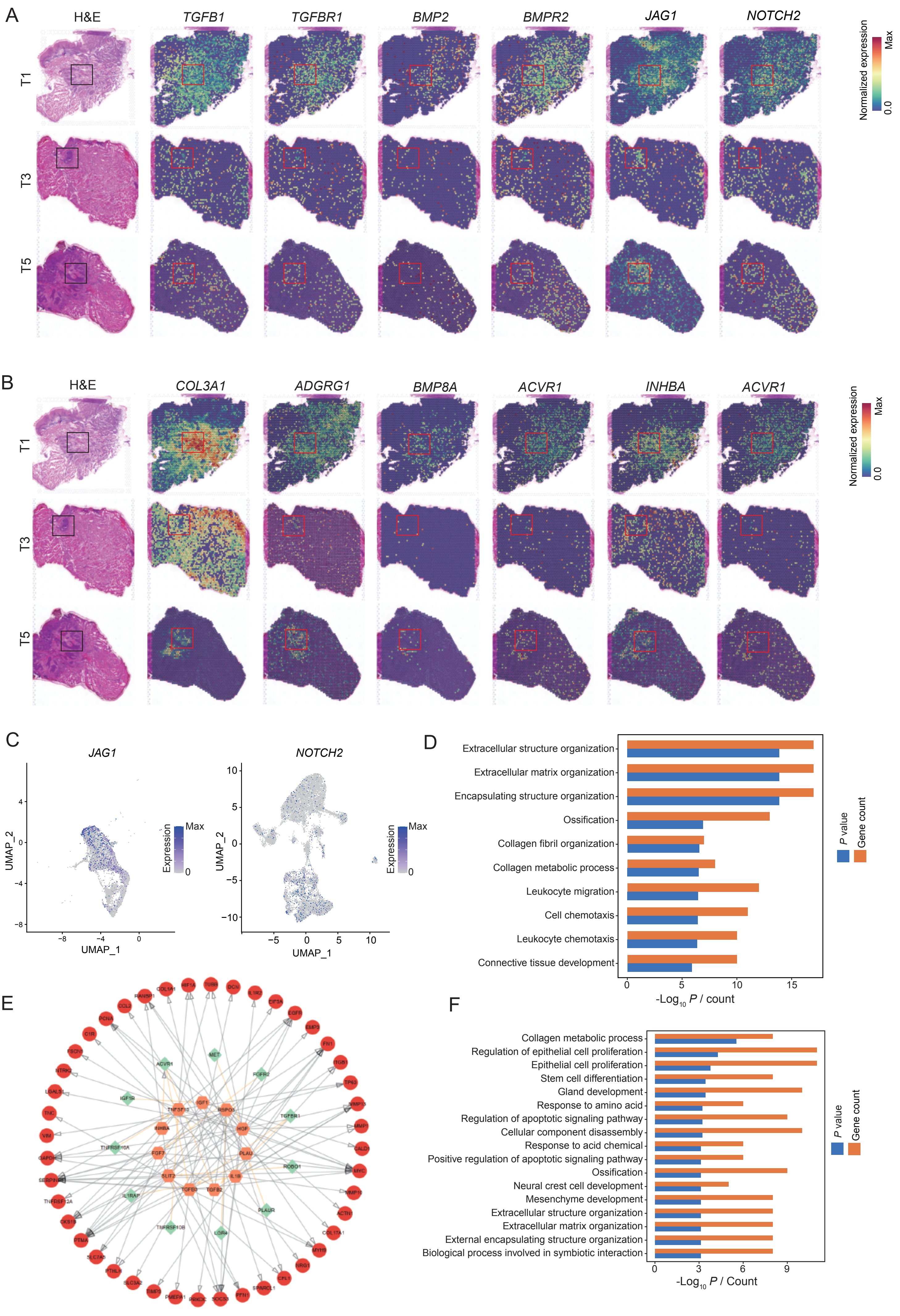
A
H&E
TGFB1
TGFBR1
BMP2
BMPR2
JAG1
NOTCH2
T1
T3
T5
Normalized expression
Max
0.0
B
H&E
COL3A1
ADGRG1
BMP8A
ACVR1
INHBA
ACVR1
T1
T3
T5
Normalized expression
Max
0.0
C
JAG1
NOTCH2
UMAP_2
UMAP_1
Expression
Max
0
D
Extracellular structure organization
Extracellular matrix organization
Encapsulating structure organization
Ossification
Collagen fibril organization
Collagen metabolic process
Leukocyte migration
Cell chemotaxis
Leukocyte chemotaxis
Connective tissue development
P value
Gene count
-Log10 P / count
E
F
Collagen metabolic process
Regulation of epithelial cell proliferation
Epithelial cell proliferation
Stem cell differentiation
Gland development
Response to amino acid
Regulation of apoptotic signaling pathway
Cellular component disassembly
Response to acid chemical
Positive regulation of apoptotic signaling pathway
Ossification
Neural crest cell development
Mesenchyme development
Extracellular structure organization
Extracellular matrix organization
External encapsulating structure organization
Biological process involved in symbiotic interaction
P value
Gene count
-Log10 P / Count
